# MAEPD: A Foundation Model for Distributed Acoustic Sensing Signal Recognition via Masked Autoencoder Pre-Training and Adapter-Based Prompt Tuning

**DOI:** 10.3390/s26072057

**Published:** 2026-03-25

**Authors:** Kun Gui, Hongliang Ren, Shang Shi, Jin Lu, Changqiu Yu, Quanjun Cao, Guomin Gu, Qi Xuan

**Affiliations:** 1 Institute of Cyberspace Security, Zhejiang University of Technology, Hangzhou 310023, China; 211123030003@zjut.edu.cn (K.G.); 21123030027@zjut.edu.cn (S.S.); xuanqi@zjut.edu.cn (Q.X.); 2 Binjiang Institute of Artificial Intelligence, Zhejiang University of Technology, Hangzhou 310056, China; 3 College of Computer Science and Technology, Zhejiang University of Technology, Hangzhou 310023, China; lujin@zjut.edu.cn (J.L.); ggm@zjut.edu.cn (G.G.); 4 Information Engineering School, Hangzhou Dianzi University, Hangzhou 310018, China; cqyu@hdu.edu.cn; 5 College of Information Engineering, Zhejiang University of Technology, Hangzhou 310023, China; qjcao@zjut.edu.cn

**Keywords:** distributed acoustic sensing (DAS), DAS signal recognition method, foundation model, MAE (masked autoencoder), parameter-efficient fine-tuning, gait recognition

## Abstract

Artificial intelligence (AI) algorithms enhance distributed acoustic sensing (DAS) signal interpretation by leveraging large-scale acoustic data. However, heterogeneous deployment environments hinder model generalization ability and exacerbate label scarcity. To overcome these challenges, we propose MAEPD, a foundation model for DAS signal recognition trained via masked autoencoder pre-training on large-scale, unlabeled DAS data collected from diverse domains. The pre-trained model is subsequently adapted to downstream tasks using adapter-based prompt tuning (APT) with only minimal labeled samples. In the DAS gait identity recognition task, with only 240 image signals per class, APT achieves 94.75% accuracy, a 4.46% improvement over full fine-tuning while updating only 2.77% of parameters. Inference latency of 2.74 ms per image meets real-time requirements. Compared to pre-training with gait data only (35.6 k samples), MAEPD improves accuracy by 3.88%, demonstrating the advantage of diverse pre-training data. The method shows robust performance across water pipe leakage, perimeter security, and public datasets, with low sensitivity to labeled data quantity. Results demonstrate an efficient and scalable solution for DAS signal recognition.

## 1. Introduction

Phase-sensitive optical time-domain reflectometry (φ-OTDR)-based distributed acoustic sensing (DAS) detects phase changes in Rayleigh backscatter along an optical fiber. These changes enable continuous, distributed measurement of acoustics, strain, and vibration [1,2,3]. By analyzing signal delay and phase, events can be precisely localized, and their dynamics can be effectively captured. DAS technology provides distributed coverage, long range, high sensitivity, real-time operation, and strong immunity to electromagnetic interference [4]. It is applied in oil and gas exploration [5,6], intelligent traffic monitoring [7,8,9], marine geophysics [10,11,12], natural earthquake monitoring [13,14], and perimeter security [15,16,17]. These applications highlight its significant potential for wider adoption.

Currently, artificial intelligence (AI) algorithms, particularly deep learning models, have significantly enhanced the automation and intelligence of DAS signal processing [18,19,20,21,22]. Early research primarily relied on feature engineering and traditional machine learning methods. For example, Wu et al. extracted frequency-domain features from pipeline monitoring signals using wavelet decomposition and combined them with a backpropagation neural network, achieving a recognition accuracy of 94.4% [23]. Pedersen et al. [11] recently applied traditional machine learning techniques to DAS data from the SHEFA-2 submarine cable, using principal component analysis (PCA) to explore signal separability and clustering to classify acoustic events such as ships, vehicles, earthquakes, and cable damage, demonstrating the potential of automated classification for maritime monitoring applications. With the development of deep learning technologies, architectures such as Convolutional Neural Networks (CNNs) [24] and Long Short-Term Memory (LSTM) networks [25] have been widely applied to DAS signal recognition tasks. For instance, Li et al. proposed a method combining CNN and LSTM for φ-OTDR fiber vibration signal recognition, effectively improving the extraction and classification of temporal features [26]. Later, the Transformer architecture [27,28] gained attention for its powerful global modeling capability. Huang et al. achieved an average recognition accuracy of 97.3% in a DAS cable intrusion detection system based on a Transformer model [29]. These studies demonstrate the significant advantages of deep learning in enhancing recognition performance for specific scenarios.

Despite these advancements, two fundamental challenges remain in practical DAS applications: insufficient model generalization and scarce labeled data [18,30]. DAS systems are often deployed in heterogeneous environments, involving varying installation conditions, geological backgrounds, operating scenarios, and interference sources, leading to significant differences in the distribution of collected signals. Models trained in controlled laboratory environments often fail to generalize well to new scenarios, and resulting in noticeable performance degradation [15,30,31]. For example, detection models for underground pipeline leaks, trained for specific soil types (such as loose sandy soil or wet farmland), show a significant decrease in accuracy when applied to different geological conditions, and traditional transfer learning methods provide only limited improvement [32,33,34]. Additionally, obtaining high-quality labeled DAS data is costly and labor-intensive, especially for sparse events such as leaks, intrusions, and seismic occurrences. Expert labeling is not only time consuming but also difficult to cover diverse operational conditions [35,36]. Therefore, improving model generalization in the presence of limited labeled data and complex environmental conditions has become a key challenge in DAS intelligent recognition research.

Transfer learning has emerged as a common strategy to address these challenges, enhancing model generalization and reducing labeling requirements by adapting models to new domains [30,31]. Shi et al. pre-trained the AlexNet network on a large-scale image dataset and fine-tuned it with DAS samples from the target domain, enabling rapid learning and classification on small-scale datasets from new scenarios [31]. Wang et al. converted one-dimensional (1D) DAS vibration signals into 2D images using Gramian angular summation fields (GASF) and fine-tuned a pre-trained ConvNeXt-Tiny network for transfer across experimental conditions, achieving recognition accuracies of 99.3% and 98.3% in single-point and dual-point perturbation experiments, respectively [37]. Zhang et al. proposed an adaptive decentralized AI approach that fine-tunes pre-trained models using unlabeled data from each domain to enhance overall generalization [30]. He et al. introduced a weighted partial domain adaptation (PDA) method for acoustic scene classification, transferring knowledge from large labeled source datasets to smaller unlabeled target datasets by establishing domain connections [38]. While these methods demonstrate the value of transfer learning, they share critical limitations [30,31,35,36]: (1) they depend on large-scale, high-quality labeled source data, which are themselves difficult to acquire [35,36]; (2) they exhibit limited generalization when source and target distributions differ substantially, as supervised pre-training assumes similar distributions between domains [30,31]; and (3) they lack interoperability across different preprocessing pipelines and signal representations commonly used in DAS applications (e.g., spectrograms, Gramian fields, spatiotemporal images) [39,40].

Self-supervised learning (SSL) offers a fundamentally different paradigm that addresses these limitations by learning representations from unlabeled data through pretext tasks that exploit the intrinsic structure of the data [36]. SSL has demonstrated remarkable success in large-scale pre-training for vision and language, with learned representations transferring effectively to diverse downstream tasks [41,42,43]. For DAS applications, SSL presents several distinct advantages: (1) elimination of labeling requirements: SSL can leverage massive unlabeled DAS datasets that are abundantly available from continuous monitoring operations, learning meaningful features without manual annotation [36]; (2) enhanced generalization: SSL pre-training captures domain-agnostic features that transfer robustly across different deployment scenarios, a critical property given the heterogeneity of DAS measurement environments [18]; (3) multi-representation learning: SSL can learn from diverse DAS data representations (e.g., GASF, Mel spectrograms, time–frequency, and spatiotemporal 2D signals) simultaneously, enabling cross-modal feature extraction without requiring separate annotations for each representation [36]; and (4) task independence: The features learned by SSL are not tied to specific classification tasks, allowing flexible adaptation to various downstream applications through efficient fine-tuning [41,42,43]. Despite these advantages, the application of self-supervised learning (SSL) in DAS signal recognition remains in its early stages. Recent studies have begun to explore the use of SSL for specific DAS tasks, such as denoising and event feature extraction using masked autoencoders (MAE) [44,45,46]. However, these efforts are confined to single-task scenarios and have not yet proposed an effective general-purpose foundation model for multi-domain DAS signal recognition. Additionally, existing research has not fully utilized large-scale, multi-domain unlabeled data for pre-training and lacks methods for efficient parameter fine-tuning to adapt to diverse downstream tasks.

To fill this gap, this study proposes a DAS signal recognition framework based on self-supervised learning and parameter-efficient fine-tuning. The central hypothesis is that self-supervised pre-training on large-scale, multi-domain unlabeled DAS data can learn robust semantic representations of DAS signals. These representations can then be effectively adapted to diverse downstream tasks through parameter-efficient fine-tuning with limited labeled samples. Based on this hypothesis, this study pursues three main objectives. First, to construct a DAS-specific foundation model through masked autoencoder pre-training on large-scale unlabeled DAS data. Second, to develop an efficient adaptation strategy that reduces the number of trainable parameters and computational cost while maintaining strong recognition performance. Third, to assess the performance of the proposed framework on several representative downstream DAS tasks from the perspectives of recognition accuracy, parameter efficiency, and generalization across different scenarios.

The main contributions of this work are summarized as follows. (1) We develop MAEPD, the first foundation model specifically designed for DAS signal recognition. Unlike existing MAE-based methods, which are mainly developed for natural images or general-purpose visual pre-training, MAEPD is pre-trained on a large-scale multi-domain unlabeled DAS dataset containing 635,860 samples. These samples cover diverse scenarios, including gait recognition, perimeter intrusion detection, pipeline leakage monitoring, earthquake sensing, and whale vocalization signals. This design enables the model to learn deep semantic representations from heterogeneous DAS data. It also provides a unified pre-trained backbone for downstream DAS tasks. (2) We propose a novel Adapter-Prompt Tuning (APT) strategy for efficient downstream adaptation. Different from conventional parameter-efficient fine-tuning methods that use either adapters or prompt vectors alone, APT integrates both mechanisms into a unified framework. By freezing the pre-trained encoder and optimizing only a small number of task-specific parameters, the proposed method achieves effective adaptation under limited labeled data. At the same time, it substantially reduces computational cost. (3) We conduct experiments on several representative downstream DAS tasks, including gait recognition, pipeline leakage detection, perimeter security event classification, and public benchmark datasets. The results show that the proposed framework maintains stable performance across different application scenarios. In particular, in the gait recognition task, APT achieves an accuracy of 94.75% using only 240 samples per class while updating merely 2.77% of the model parameters. This performance is superior to that of conventional supervised learning methods. These results validate the effectiveness of the proposed framework.

## 2. DAS Principle and Signal Pre-Processing

**Figure 1:**
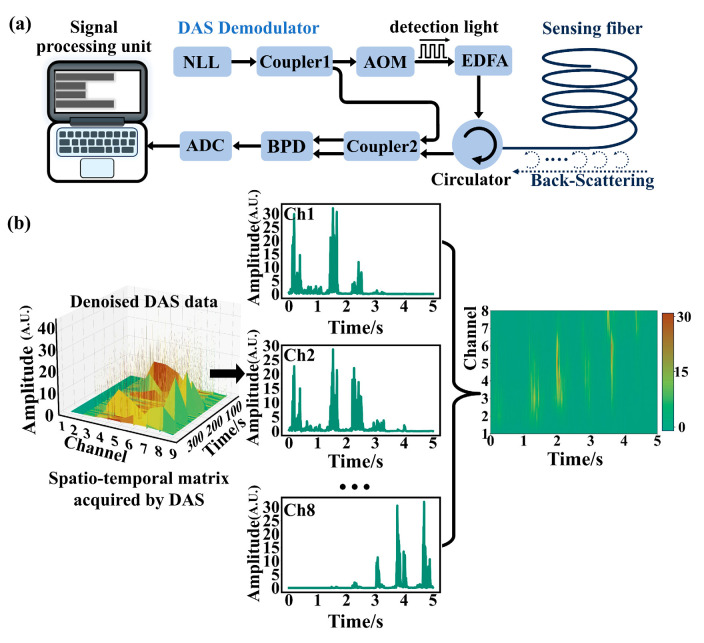
(**a**) The main block diagram of the DAS system. NLL (Narrow Linewidth Laser): Central Wavelength: 1550 nm, Output Power: 13 dBm, Linewidth: 3 kHz; AOM (Acousto-Optic Modulator): Rise Time: 20 ns, Frequency Shift:200 MHz, Drive Power: 2 W, EDFA (Erbium-Doped Fiber Amplifier): Operating Wavelength: 1550 nm, Input Peak Average Optical Power: −30 dBm, Output Peak Power: 23 dBm, Pulse Width: 5–100 ns, Repetition Frequency: 2 kHz; BPD (Balanced Photodetector): Bandwidth: 350 MHz, Saturation Input Optical Power: −8.24 dBm. (**b**) Pre-processing pipeline for Spatiotemporal signal: Raw 1D amplitude signals from 8 channels (vertical axis) over a 5 s window (horizontal axis) are stacked to form a 2D spatiotemporal image of size 224 × 224 pixels.

Figure 1a shows the schematic of the φ-OTDR-based DAS system. The DAS system comprises three core components: the sensing fiber, the DAS demodulator, and the signal processing unit. It detects changes in fiber vibration and its surrounding environment by analyzing Rayleigh backscattering light transmitted through the optical fiber. When external vibrations act on a section of the sensing fiber, the relative positions of Rayleigh scattering centers shift, resulting in a local phase change. By monitoring these phase variations, the system can accurately capture disturbances such as vibrations and acoustic events near the fiber.

The working principle of the φ-OTDR-based DAS system is as follows. A narrow linewidth laser (NLL) emits a continuous optical wave at a wavelength of 1550 nm. This light is split into two paths by a 2 × 2 coupler (Coupler 1). One path is directed to an acousto-optic modulator (AOM), which, driven by an AOM driver (operating at 200 MHz, with an RF power of <2 W, a switch ratio of 60 dB, and a rise/fall time of <10 ns), modulates the continuous wave into optical pulses. These pulses are subsequently amplified by an erbium-doped fiber amplifier (EDFA) and then launched into the sensing fiber of length L via an optical circulator. The sensing fiber is conceptually divided into *N* segments with a spatial resolution of *l*, each acting as a discrete reflector. The backscattered signals arise from the combined contribution of *M* Rayleigh backscatter centers within length *L*. As the backscattered light propagates along the fiber, the phase delay *φ* can be expressed as *φ* = *βL*, where *β* is the propagation constant of light in the fiber. When the fiber at position *z* is subjected to external disturbances, variations in fiber length (*L*), core diameter (*α*), and refractive index (*n*) induce changes in the phase of the Rayleigh backscattered light:

(1) $$\begin{array}{cc} \Delta \varphi & =\;\;\beta \Delta L+L\Delta \beta \\ & =\beta \Delta L+L(\frac{\partial \beta }{\partial n})\Delta n+L(\frac{\partial \beta }{\partial \alpha })\Delta \alpha \end{array}$$

Here ΔL, Δn and Δα represent the phase changes caused by variations in the fiber length, refractive index, and diameter at position z, respectively, due to external disturbances. Then, the Rayleigh backscattered light is routed by a circulator and mixed with a local-oscillator (LO) field tapped from the second output of coupler 1 for heterodyne detection. A balanced photodetector (BPD, bandwidth: 350 MHz) converts the optical beat into an electrical signal, which is digitized by an analog-to-digital converter (ADC) and digitally I/Q-demodulated to recover phase fluctuations induced by fiber perturbations [47]. During each pulse emission cycle, the DAS system records an OTDR trace as a function of spatial fiber position. By continuously acquiring OTDR traces over successive time intervals, a spatiotemporal matrix is formed to represent disturbance events in 2D spatiotemporal space. This matrix is then used to detect and analyze target signals, such as vibrations or acoustic waves.

In this study, we investigate signal preprocessing methods to convert raw one-dimensional (1D) DAS signals into two-dimensional (2D) image representations suitable for deep learning models. Since OTDR traces often contain background noise that degrades measurement precision and event recognition reliability, wavelet denoising is employed for preprocessing. Specifically, 1D DAS temporal signals are decomposed into multiscale frequency components via the wavelet transform. The wavelet coefficients are then thresholded to suppress noise, and the signals are reconstructed using the inverse wavelet transform. After denoising, we adopt three distinct transformation strategies to generate 2D images, each tailored to different application scenarios and aimed at enriching the diversity of the pre-training dataset. (1) Spatiotemporal stacking [31,48]: We directly stack the denoised 1D amplitude signals from multiple channels into a 2D spatiotemporal image. As illustrated in Figure 1b, the horizontal axis represents time (with 10,000 sampling points over 5 s), and the vertical axis corresponds to the channel index (eight channels in total). This representation preserves both the temporal dynamics and the spatial distribution of vibration events. (2) Short-Time Fourier Transform (STFT): For applications where frequency-domain features are more discriminative, we convert the denoised 1D signals into 2D time–frequency images using STFT [49,50]. For a single-channel DAS vibration signal *x*(*t*), after sampling we obtain a discrete sequence *x*[*n*], and its STFT is computed as:

(2) $$X(m,f)=\sum_{n}x[n]w[n-m]e^{-j\;2\pi fn}$$

where *w*[*n*] is a window function of length *N* = 128, *m* is the time index, *f* is the frequency bin. In this work, each 2 s signal segment is processed with a window length of 256 samples and 50% overlap, and the resulting time–frequency matrix is mapped to a 224 × 224 image. (3) Gramian Angular Summation Field (GASF) [37]: For tasks involving transient or non-stationary events, we employ GASF to encode the 1D time series into 2D images. Given a normalized time series $\tilde{x}=\{{\tilde{x}}_{1},{\tilde{x}}_{2},\ldots ,{\tilde{x}}_{N}\}$, the sequence is converted into angles and radii via polar coordinate mapping:

(3) $$\phi_{i}=\arccos({\tilde{x}}_{i}),\;\;\;\;r_{i}=\frac{t_{i}}{T}$$

where *t_i_* is the time index and *T* is a constant factor used to normalize the polar radius. The GASF matrix *G_i,j_* is generated by calculating the cosine of the sum of angles for each pair of points:

(4) $$G_{i,j}=\cos(\phi_{i}+\phi_{j})$$

Each entry captures the temporal correlation between two points, and the resulting two-dimensional image preserves the temporal dependencies. In this work, the GASF image is resized to 224 × 224. By incorporating these diverse 2D representations, we substantially increase the richness of the data, enabling the self-supervised pre-training model to learn robust features across multiple DAS application domains.

## 3. Proposed DAS Signal Recognition Method

**Figure 2:**
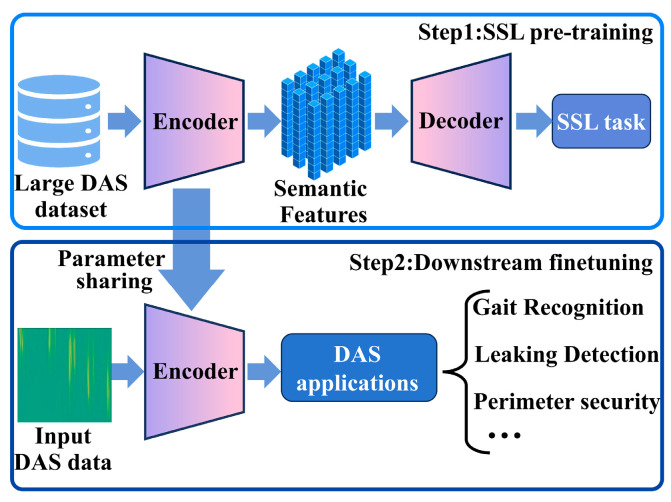
Overview of the proposed two-stage MAEPD + APT framework. Step 1 (SSL pre-training): MAE is pre-trained on a large-scale, unlabeled DAS dataset. The encoder learns semantic features by reconstructing masked image patches. Step 2 (downstream fine-tuning): The pre-trained encoder is frozen and adapted to multiple downstream tasks (e.g., gait recognition, pipeline leakage detection, perimeter security) using adapter-based prompt tuning (APT) with minimal labeled data.

To address the common challenge in DAS applications of abundant unlabeled data but scarce labeled data, we propose a two-stage framework as illustrated in Figure 2: (1) self-supervised pre-training to build a DAS-specific foundation model, subsequently followed by (2) parameter-efficient fine-tuning for downstream tasks. In the first stage (Step 1: SSL pre-training), we leverage a large-scale, unlabeled DAS dataset to train a masked autoencoder (MAE). The MAE framework consists of an encoder that extracts deep semantic representations from visible image patches and a lightweight decoder that reconstructs the original image from the encoded features. By solving this self-supervised reconstruction task, the encoder learns to capture generalizable semantic features from diverse DAS signals across multiple domains (e.g., gait, pipeline leakage, perimeter intrusion, whale vocalization, and seismic events). After pre-training, the decoder is discarded, and the encoder is retained as the MAEPD foundation model. In the second stage (Step 2: downstream fine-tuning), the pre-trained encoder is adapted to various downstream tasks, including gait recognition, pipeline leakage detection, and perimeter security event classification, using only a limited number of labeled samples. To enable efficient adaptation with minimal parameter updates, we employ a parameter-efficient fine-tuning technique, adapter-based prompt tuning (APT) [51,52], which introduces lightweight trainable modules (adapters and visual prompts) while keeping the encoder backbone frozen. This enables efficient parameter sharing across multiple DAS applications with minimal labeled data and computational cost. The following sections will detail the construction of the MAEPD foundation model and the APT-based fine-tuning approach.

### 3.1. MAEPD: A DAS-Specific Foundation Model Based on Self-Supervised Pre-Training

#### 3.1.1. Construction of the Pre-Training DAS Dataset

To support self-supervised pre-training of the MAEPD foundation model, we constructed a large-scale and diverse dataset comprising 635,860 2D image samples, each resized to 224 × 224 pixels. As summarized in Table 1, this dataset integrates data from six distinct sources, covering multiple DAS application scenarios including gait recognition, pipeline leakage, perimeter intrusion, whale vocalization, and seismic events. The data originate from both self-conducted laboratory experiments and publicly available repositories [53,54,55]. To enrich representation diversity, three different preprocessing techniques: spatiotemporal stacking, short-time Fourier transform (STFT), and Gramian angular summation field (GASF) were applied to convert raw 1D signals into 2D image formats, as detailed in Section 2 and Appendix A.1, Appendix A.2 and Appendix A.3.

**Figure 3:**
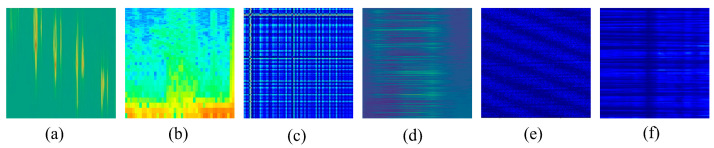
Representative 2D image samples from the six datasets used for MAEPD pre-training (see Table 1). (**a**) 2D spatiotemporal gait signals collected in the experiment. (**b**) 2D time–frequency pipeline leakage signals collected in the experiment. (**c**) 2D Gramian angular summation field (GASF) perimeter intrusion signals collected in the experiment. (**d**) 2D spatiotemporal perimeter intrusion signals from a publicly available dataset [53]. (**e**) 2D spatiotemporal whale vocalization signals from a publicly available dataset [54]. (**f**) 2D spatiotemporal seismic signals from a publicly available dataset [55].

Specifically, the self-collected datasets include the following. (1) Gait signals: 35,637 spatiotemporal images acquired from walking trials on a wooden floor instrumented with fiber-optic cables, with variations in walking speed, footwear, and background noise levels; (2) various 1D pipeline-leakage signals (PLS) were collected using a DAS-based pipeline-leakage detection system and converted via STFT into 260,154 2D time–frequency images at 224 × 224 resolution; the dataset and acquisition system are described in Appendix A.1; (3) 1D perimeter-intrusion signals acquired with a DAS-based system were converted via GASF into 150,170 2D images at 224 × 224 resolution; further details are provided in Appendix A.2. The publicly available datasets include the following. (1) Perimeter Intrusion signals: 6000 spatiotemporal images from Beijing Jiaotong University [53]; (2) Whale Vocalization signals: 171,302 spatiotemporal images recorded by a seabed DAS system [54], capturing fin whale and blue whale vocalizations; and (3) Seismic signals: 12,597 spatiotemporal images from the FORESEE project [55], including natural and anthropogenic seismic events. Further details are provided in Appendix A.3. Figure 3 displays representative 2D image samples from each of the six datasets listed in Table 1, illustrating the distinct visual characteristics of different DAS applications and preprocessing methods. Importantly, the pre-training dataset is completely disjoint from the fine-tuning and test datasets used in downstream tasks at the sample level, with no sample overlap.

#### 3.1.2. MAE Pre-Training Architecture and Settings

**Figure 4:**
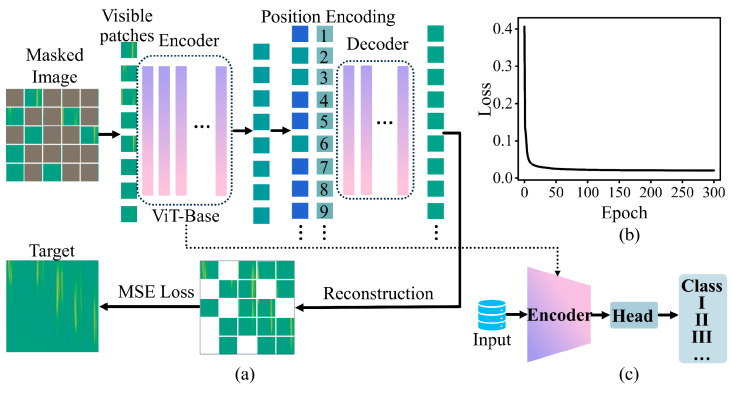
MAEPD pre-training architecture and results. (**a**) The framework architecture of a foundational model for DAS signal recognition, termed MAE-based pre-training on DAS signals (MAEPD). (**b**) The training loss curve during the MAEPD pre-training phase. (**c**) After the MAE pre-training is completed, its encoder is frozen and saved for use in downstream classification tasks.

Figure 4a illustrates the pre-training model built on the MAEPD framework. MAE adopts an asymmetric encoder–decoder architecture and achieves efficient self-supervised and representation learning through a masking-and-reconstruction task. The framework comprises an encoder and a decoder. The encoder is a ViT that operates only on unmasked image patches to extract deep semantic features from visible regions; the decoder reconstructs the full image from the latent representations. The encoder and the decoder are stacks of transformer encoder blocks, each consisting of alternating multi-head self-attention (MHSA) layers and multi-layer perceptron (MLP) blocks. Layer normalization is applied before each block, and residual connections are added afterwards. Concretely, a DAS image is partitioned into *m* fixed-size patches: $\left\{I_{j}\in {\mathbb{R}}^{3\times h\times w}\left|j\in \mathbb{N},1\leq j\leq m\right.\right\}$, where *I_j_* denotes the *j*-th patch, and *h*, *w* are the patch height and width, respectively. Let $I_{j}^{v}$ be the visible patches and $I_{j}^{n}$ the randomly masked patches, visible patches $I_{j}^{v}$ are embedded into a *d*-dimensional space:

(5) $$e_{0}^{j}=\mathrm{Embed}(I_{j}^{v})\;\;\;e_{0}^{j}\in {\mathbb{R}}^{d},j=1,2,\ldots m$$

(6) $$E_{i}^{v}=\{e_{i}^{j}\in {\mathbb{R}}^{d}\left|j\in \mathbb{N},\right.1\leq j\leq m\}$$

Here, $e_{0}^{j}$ denotes the *d*-dimensional embedding of the *j*-th visible image patch, and $E_{i}^{v}$ denotes the set of *d*-dimensional embeddings of all visible patches at encoder layer *i*. A class token *x_i_* is concatenated with the patch embeddings, after which positional encoding is added to preserve spatial information. This yields the complete input sequence, which is then fed into an *N*-layer encoder:

(7) $$[x_{i},E_{i}^{v}]\;=\;L_{i}([x_{i-1},E_{i-1}^{v}])\;\;\;i=1,2,\ldots ,N$$

Here, *L_i_* denotes the *i*-th encoder layer. The class token at this layer is represented by a *d*-dimensional embedding *xᵢ*∈ℝ*^d^*. Concatenating *xᵢ* and $E_{i}^{v}$ along the leading (sequence) dimension yields $[x_{i},\;\;E_{i}^{v}]\in {\mathbb{R}}^{(1+m)\times d}$, where *m* is the number of visible patches. After processing by *N* encoder layers, we obtain the semantic representation of visible patches $E_{N}^{v}$. Preserving the original patch order, $E_{N}^{v}$ is concatenated with the mask tokens *E^n^* to form the complete sequence $\left[E_{N}^{v},\;E^{n}\right]$. Positional encodings are then re-added to this sequence (including both encoded visible patches and mask tokens) to retain spatial structure. The decoder consumes this sequence, and its output is projected back to pixel space through a linear layer to produce the reconstructed image. During training, the reconstruction loss is computed only over the masked patches using mean squared error (MSE):

(8) $$L_{r}=\frac{1}{M}{\sum_{i\in M}\left\|x_{i}-{\hat{x}}_{i}\right\|}^{2}$$

where M is the set of pixels within the masked regions, and *x_i_* and ${\hat{x}}_{i}$ are the values of the original and reconstructed images at pixel *i*, respectively. Restricting the loss to masked regions encourages the model to focus on this challenging prediction task.

The detailed hyperparameters and experimental setup for MAEPD pre-training are summarized in Table 2. During MAEPD pre-training, all input images were standardized to 224 × 224 pixels via random cropping and resizing, with a random masking ratio of 75% [56]. We used AdamW with an initial learning rate of 0.001 and a cosine-annealed schedule, a mini-batch size of 16, and trained for 300 epochs. The upstream pre-training experiments were performed on an Intel Core i5-12600KF CPU and an NVIDIA GeForce RTX 4070 Ti GPU, with the total wall-clock training time amounting to approximately 350 h and 20 min. As shown in Figure 4b, the loss curve plateaus around the 200th epoch and the final training loss reaches 0.020, indicating full convergence. This demonstrates that the MAE paradigm converges reliably for 2D DAS image reconstruction and effectively captures the semantic structure of DAS images. After pre-training (Figure 4c), the lightweight decoder is discarded and only the encoder is retained as the pre-trained backbone for feature extraction or fine-tuning across downstream DAS classification tasks.

### 3.2. Adapter-Based Prompt Tuning (APT) of the Pre-Trained Model

**Figure 5:**
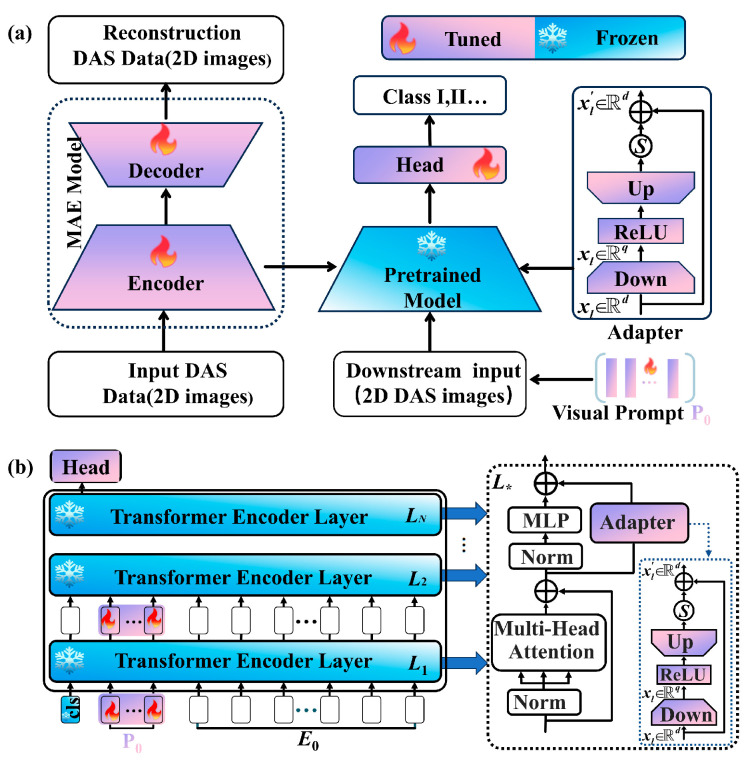
(**a**) APT for parameter-efficient fine-tuning. (**a**) The pre-trained MAEPD encoder is frozen, and a task-specific classification head is attached. Learnable visual prompt vectors and adapter modules are introduced. (**b**) Detailed APT architecture: for each transformer encoder layer, learnable prompt vectors are prepended to the input sequence. A lightweight adapter is inserted in parallel. Only the prompts, adapters, and classification head are updated during fine-tuning. L* denotes each transformer encoder layer.

We use ViT-Base as the pre-trained backbone. Given its scale and numerous parameters, conventional FFT, which updates most or all weights, requires substantial training time, and compute [57]. We therefore adopt a parameter-efficient approach, APT for downstream fine-tuning [58]. Learnable visual prompt vectors are prepended to the model input [51], and lightweight trainable adapters are inserted between the frozen layers of the pre-trained backbone [52]. This configuration enables rapid and robust adaptation to downstream tasks while keeping the backbone largely frozen. For downstream recognition, a task-specific classification head is attached to the pre-trained encoder, as illustrated in Figure 5a. During fine-tuning, the encoder remains frozen while optimization is restricted to the adapters inserted in each encoder layer, the learnable visual prompts embedded in the input sequence, and the classification head. At inference, with the backbone, prompts, adapters, and classification head fixed, the model enables fast, efficient, and accurate recognition on unseen samples.

APT, as illustrated in Figure 5b, lightweight trainable adapter module is inserted into every layer of the encoder. Each adapter follows a bottleneck design, consisting of a down-projection layer $W_{Down}\in {\mathbb{R}}^{d\times q}$, an up-projection layer $W_{Up}\in {\mathbb{R}}^{q\times d}$, and a ReLU activation function placed between the two projection layers to introduce nonlinearity. Let *q* denote the bottleneck dimension, with *q* < *d*. Specifically, the input features are first mapped to a *q*-dimensional low-dimensional bottleneck space, then transformed nonlinearly by the ReLU function, and finally projected back to the original dimension through the up-projection layer. This bottleneck module is integrated into the backbone network via a residual connection with a scaling factor *S*, thereby preserving model stability while achieving parameter-efficient feature adaptation. For a given input feature $x_{l}\in {\mathbb{R}}^{d}$, the output feature $x_{l}^{\prime }$ after passing through the adapter module can be expressed as:

(9) $$x_{l}^{\prime }=S\cdot (\mathrm{ReLU}(\mathrm{LN}(x_{l})\cdot W_{Down})\cdot W_{Up})+x_{l}$$

APT also inserts learnable prompt vectors into the input of each transformer encoder layer. These vectors have the same dimensionality as the patch embeddings *E_i_* (dimension *d*). Specifically, for the (*i* + 1)-th transformer encoder layer *L_i_*_+1_, a set of prompt vectors P*_i_* is added to its input:

(10) $$P_{i}={\{p}_{i}^{k}\in {\mathbb{R}}^{d}\left|k\in \mathbb{N},1\leq k\leq m\right.\}\;\;\;i\;=\;0,\;1,\;2,\;\ldots \;,\;N$$

Let $P_{i}\in {\mathbb{R}}^{m\times d}$ denote the *m* prompt vectors inserted at the input of the *i*-th transformer encoder layer. The concatenation $\left[x_{i\;},\;P_{i\;}\;,\;E_{i\;}\right]$ forms the input to the layer, where *xᵢ*∈ℝ*^d^* is the *d*-dimensional class-token embedding at layer *i*, and $E_{i}$ denotes the set of *d*-dimensional patch embeddings at the same layer. The resulting sequence is first passed through an MHSA and then an MLP:

(11) $$\left[x_{i}^{\prime },\;P_{i}^{\prime },\;E_{i}^{\prime }]=\mathrm{MHSA}(\mathrm{Norm}([x_{i},\;P_{i},\;E_{i}]))+[x_{i},\;P_{i},\;E_{i}\right]$$

(12) $$[x_{i}^{\prime \prime },\;P_{i}^{\prime \prime },\;E_{i}^{\prime \prime }]=\mathrm{MLP}(\mathrm{Norm}([x_{i}^{\prime },\;P_{i}^{\prime },\;E_{i}^{\prime }]))$$

$\left[x_{i}^{\prime },\;P_{i}^{\prime },\;E_{i}^{\prime }\right]$ and $\left[x_{i}^{\prime \prime },\;P_{i}^{\prime \prime },\;E_{i}^{\prime \prime }\right]$ denote the outputs of the MHSA and MLP blocks of the *i*-th transformer encoder layer, respectively. Moreover, the input $\left[x_{i}^{\prime },\;P_{i}^{\prime },\;E_{i}^{\prime }\right]$ is also routed through an adapter branch, whose output is given by:

(13) $$\left[x_{i}^{\prime \prime \prime },\;P_{i}^{\prime \prime \prime },\;E_{i}^{\prime \prime \prime }]=S^{\prime }\cdot (\mathrm{ReLU}(W_{Down}\cdot ([x_{i}^{\prime },\;P_{i}^{\prime },\;E_{i}^{\prime }]))\cdot W_{Up})+[x_{i}^{\prime },\;P_{i}^{\prime },\;E_{i}^{\prime }\right]$$

Here, $S^{\prime }$ denotes the scaling factor applied to the output of the adapter module. The output of the *i*-th transformer encoder layer is:

(14) $$\left[x_{i+1}^{*},\;\_\_,\;E_{i+1}^{*}]\;=\;[x_{i}^{\prime \prime \prime },\;P_{i}^{\prime \prime \prime },\;E_{i}^{\prime \prime \prime }]+[x_{i}^{\prime \prime },\;P_{i}^{\prime \prime },\;E_{i}^{\prime \prime }\right]$$

(15) $$y=\mathrm{Head}(x_{N})$$

After processing by *N* transformer encoder layers, an MLP maps the class token from the *N*-th layer to the predicted class probability distribution y. Introducing visual prompt vectors into each transformer encoder layer increases the input dimensionality and consequently the output dimensionality. Because visual prompt vectors are injected from the first to the last layer, the output dimensionality would grow with depth. Therefore, as shown in Equation (14), we remove the prompt vectors from the output of every encoder layer to keep the input dimensionality consistent across layers. After designing and inserting the visual prompt vectors and the lightweight trainable adapter modules, both components are optimized to ensure strong adaptability to downstream tasks. For downstream classification, fine-tuning uses the cross-entropy loss function as the objective:

(16) $$\mathrm{Loss}=-\frac{1}{N}\sum_{n=1}^{N}\sum_{c=1}^{M}y_{c}^{\left(n\right)}\log({\hat{y}}_{c}^{\left(n\right)})$$

Here, *N* denotes the number of samples and *M* the number of classes. $y_{c}^{\left(n\right)}$ is the ground-truth probability that the *n*-th sample belongs to class *c*, while ${\hat{y}}_{c}^{\left(n\right)}$ is the model predicted probability for the sample belonging to class *c*.

## 4. Experiment Scenes and Dataset

This section presents the experimental setup and datasets used to validate the proposed MAEPD + APT method. The validation is conducted through multiple downstream tasks, with a primary focus on gait-based identity recognition using DAS signals acquired from indoor floor walking. Additional downstream tasks include simulated pipeline leakage event detection, perimeter intrusion event classification, and event recognition on a publicly available perimeter security dataset [53]. It is important to note that all downstream datasets used for fine-tuning and evaluation are completely disjoint from the pre-training dataset at the sample level.

### 4.1. Gait Experimental Scene and Dataset

Gait, as a highly distinctive biometric feature, reflects the movement patterns of the human body during walking [59]. Through the measurement and analysis of these dynamic features, gait recognition technology can achieve both identity verification and behavior monitoring, offering broad application prospects in fields such as biometric authentication, health assessment, and security monitoring [60,61]. Zhou et al. [60] deployed optical cables along a corridor and used a DAS system to capture gait signals from pedestrians, applying a convolutional long short-term memory (ConvLSTM) network for recognition, achieving an accuracy of 81.35%. Shi et al. [61] laid optical fibers on the ground, precisely capturing vibration signals caused by gait through DAS, and developed a dual YOLO model to analyze stride length and frequency features, enabling identity recognition and footstep localization for three subjects with an accuracy of approximately 86.0%. These studies collectively validate the advantages and potential of DAS technology for large-scale gait recognition applications.

**Figure 6:**
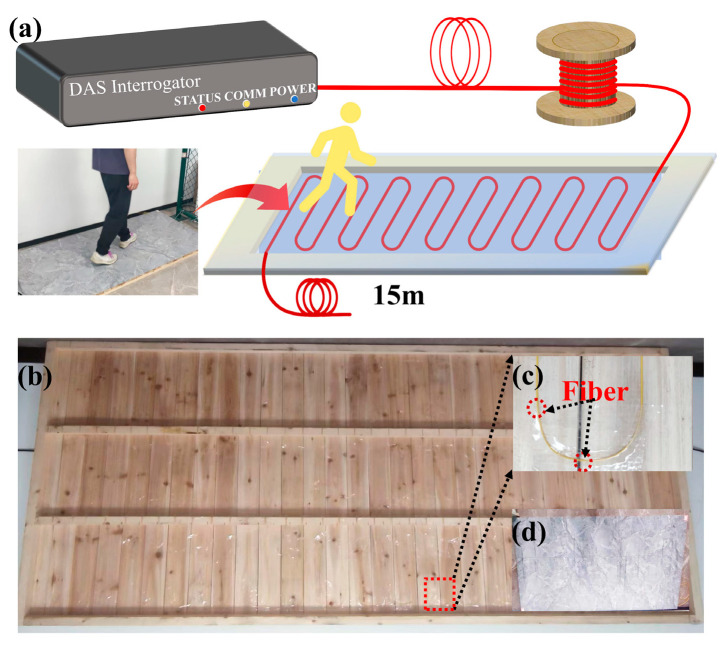
(**a**) An experimental setup diagram for gait signal acquisition via a DAS system. A single-mode optical fiber with a protective sheath is laid in an S-shaped configuration on the backside of the wooden floor. The testing system’s fiber starts from the DAS demodulator, coiling along a certain distance (less than 5 km), and eventually connects to the optical fiber laid on the wooden floor. (**b**) Photograph of the wooden floor with optical fiber arranged on the backside. (**c**) Magnified image of the highlighted area in (**b**). (**d**) Photograph of the front side of the wooden floor.

As shown in Figure 6a, we designed and implemented an indoor DAS gait signal measurement system. This system uses G652.D single-mode optical fiber, with a protective sheath, laid on the backside of a wooden floor and connected to a DAS demodulator via optical fiber of a certain length. During the experiment, the tester walks on a wooden floor, the underside of which is equipped with optical fibers, with environmental background noise maintained at typical indoor levels. The DAS system has a detection distance of 5 km, 960 channels, a spatial resolution of 5 m, a gauge length of 5 m, and a sampling rate of 20 kS/s. Figure 6b–d show photographs of the front and back sides of the wooden floor and the fiber installation. The optical fiber is routed in an S-shaped configuration along the short edge of the floor, mounted on wooden battens at a height of 3 cm, and securely bonded using adhesive. The installation area measures 1 m × 2 m, with a 5 cm spacing between adjacent parallel fibers. The total length of the fiber on the backside of the floor is approximately 40 m, corresponding to 8 channels.

Table 3 presents the gait-related physiological parameters of seven volunteers who participated in DAS gait signal acquisition, ensuring that the data includes subjects with diverse gender, and weight. To enhance the data diversity for upstream pre-training, the first six participants in Table 3 wore different types of footwear (e.g., sneakers and slippers) and walked in three different speeds: slow, normal, and fast. Additionally, the fiber length between the DAS demodulator and the floor fiber was varied (0.35 km, 2 km and 3.45 km), as well as different background noise levels in the indoor environment (16 dB, 35 dB and 70 dB). An additional set of 1000 samples was collected when Testers I and II walked simultaneously on the floor. After several days of data collection, a total of 35,637 224 × 224 spatiotemporal gait signals were obtained, with each signal lasting 5 s and containing 8 channels. These data were used for the upstream pre-training of the MAEPD model.

Building on this, the newly collected gait dataset from the DAS system was used for fine-tuning and evaluating the downstream tasks, independently of the MAEPD pre-training dataset. The dataset includes data from the first four volunteers listed in Table 4, each contributing gait data collected while wearing two types of shoes (sneakers and slippers). For each shoe type, data were recorded across three walking statuses, slow, normal, and fast, with an equal number of data samples obtained for each walking mode. In total, 5040 224 × 224 spatiotemporal gait signals were collected, each lasting 5 s and containing 8 channels. The dataset is organized into 8 categories, with each class representing a volunteer wearing a specific shoe type, as shown in Table 4. Each class contains 630 samples, which are further split into a training set (240 samples), a validation set (90 samples), and a test set (300 samples) for downstream tasks.

**Figure 7:**
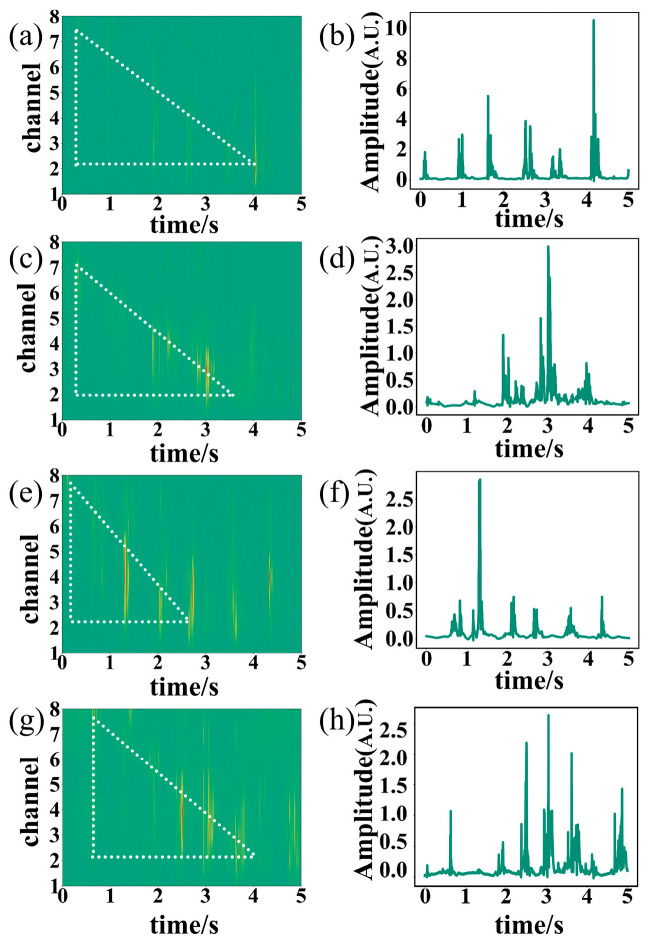
Several typical DAS gait 2D spatiotemporal and 1D amplitude signals: (**a**) 2D spatiotemporal images of Tester III walking slowly while wearing slippers; (**c**) Tester IV walking normally while wearing sneakers; (**e**) Tester IV walking at a fast pace while wearing slippers; (**g**) Tester VII walking normally while wearing sneakers. Panels (**b**,**d**,**f**,**h**) show the 1D amplitude signals obtained from Channel 3 corresponding to (**a**,**c**,**e**,**g**), respectively. The dashed lines in (**a**,**c**,**e**,**g**) represent the fitted lines through the center of each stride, used to estimate walking speed.

The testers walked on a wooden floor wearing sneakers and slippers, with vibration pressure transmitted indirectly through the floor to the optical fiber, thus avoiding potential physical damage caused by direct contact with the fiber. Figure 7 shows representative 1D amplitude gait signals and 2D spatiotemporal gait signals obtained from testers wearing different shoes and adopting different walking statuses. Figure 7a displays the 2D spatiotemporal signal of Tester III, the heaviest participant, walking slowly while wearing slippers. Figure 7c,e show the corresponding signals of Tester IV, the second lightest participant, walking normally while wearing sneakers and walking quickly while wearing slippers, respectively. Figure 7g presents the signal of Tester VII, the lightest female participant, walking normally while wearing sneakers. Figure 7b,d,f,h show the 1D amplitude signals from Channel 3 corresponding to Figure 7a,c,e,g, respectively. These results reveal a significant impact of body weight on signal intensity, with heavier subjects producing stronger gait signals. Slippers, being softer and offering better cushioning than sneakers, result in slightly weaker signals. In the 2D spatiotemporal images, distinct walking patterns can be identified. For instance, Figure 7a,c,e display walking at different speeds with a consistent stride, respectively. By fitting a line to the center of each stride across channels and calculating the slope with respect to the time axis, the walking speed can be estimated. In the experiment, the first four testers listed in Table 3 were instructed to maintain a constant stride while walking straight across a 2 m detection area, with their time recorded by a stopwatch to calculate actual walking speed. Simultaneously, DAS spatiotemporal signals were used to estimate walking speed, as described in Figure 7a,c,e. This procedure was repeated 20 times for each subject across fast, normal, and slow walking speeds, with the arithmetic mean calculated for each condition. The estimated values were then compared to the actual values, as shown in Table 5. The results demonstrate that the estimated walking speeds closely match the actual values.

### 4.2. Other Experimental Scenes and Datasets

In addition to the gait recognition task, we evaluate the proposed MAEPD + APT method on three other DAS downstream tasks with distinct application scenarios and signal characteristics: water pipe leakage detection, perimeter intrusion detection, and a public perimeter security dataset. These datasets are completely disjoint from the pre-training dataset at the sample level, ensuring unbiased evaluation. Detailed descriptions of each experimental setup, including sensor configurations, event types, and data acquisition procedures, are provided in Appendix A.1, Appendix A.2 and Appendix A.3, while the dataset splits for downstream fine-tuning and evaluation are summarized in Table 6. Specifically, the water pipe leakage dataset comprises 1D signals collected from a laboratory pipeline system converted into 224 × 224 time–frequency images using STFT, covering four event types (leakage, no leakage, hammering, cutting) with 50/100/500 samples per class for training/validation/testing. The perimeter intrusion dataset includes 1D signals from fence and ground intrusions transformed into 224 × 224 GASF images, covering five event types (shaking, UAV intrusion, biting, trampling, cutting) with 25/100/300 samples per class. The public perimeter security dataset [53] consists of spatiotemporal 2D images (224 × 224) from six event types (shaking, digging, knocking, walking, watering, background).

## 5. Results and Analysis

### 5.1. Ablation Study and Analysis on Gait Recognition

For the gait recognition task on the wooden floor, this study fine-tunes the pre-trained MAEPD encoder using the APT method described in Section 3. The main parameters of the encoder are kept frozen, while only the visual prompt vectors, the adapter modules, and the classification head are optimized during fine-tuning. To comprehensively evaluate the performance of the method, we set up two categories of baseline methods for comparison: (1) MAEPD-based fine-tuning methods, including visual prompt tuning (optimizing only the input prompt vector, also referred to as VPT) [51], adapter tuning (AT, optimizing only the adapter modules) [52], full fine-tuning (FFT, updating all model parameters), and linear probing (LP, freezing the encoder and training only the linear classifier) [58]; (2) traditional supervised learning models, including convolutional neural network (CNN) and transformer-based architectures such as MobileNetV2, ResNet-18, ConvNeXt-Tiny, and ViT-Base. Since the upstream pre-training is conducted on unlabeled 2D DAS data, these supervised learning models are trained on the labeled downstream dataset (Table 4) for fair comparison.

Hyperparameters for all methods were selected via grid search on the validation set (see Table 7 for search ranges). The learning rate is scaled according to the linear scaling rule: learning rate = base learning rate × batch size/256, with a fixed batch size of 16. The base learning rate search ranges are [5 × 10^−2^, 5] for VPT, AT, and APT methods, and [1 × 10^−4^, 5 × 10^−3^] for FFT, LP, and the traditional supervised models. The configuration yielding the highest validation accuracy was then used for final evaluation. All reported performance metrics (accuracy, precision, recall, F1-score, training time, and inference latency) are obtained exclusively on the held-out test set. All downstream fine-tuning experiments were implemented in PyTorch 2.1.0 and executed on a computing platform equipped with an Intel Core i9-14900KF processor and an NVIDIA GeForce RTX 4080 Super GPU.

**Figure 8:**
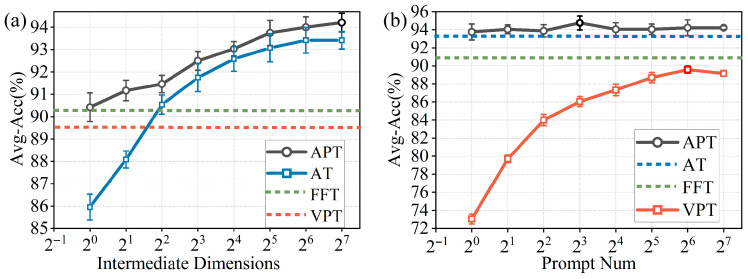
(**a**) Average test accuracy vs. intermediate dimension of the adapter across four fine-tuning methods (prompt vector number fixed at 64). (**b**) Average test accuracy vs. number of visual prompt vectors across four fine-tuning methods (intermediate dimension of adapter set to 128). Error bars represent standard deviations over five independent runs.

The experiment first evaluated the performance of four fine-tuning methods on the gait recognition task. Each experiment was independently run five times, and the average of the results was taken as the final performance metric. The dataset composition is shown in Table 4. Figure 8a illustrates the recognition accuracy versus the intermediate dimension of the adapter. For the AT method (represented by the blue curve in the figure), when the intermediate dimension is increased to 128, the accuracy saturates at 93.54%. The APT method used in this study builds upon the VPT framework (with 64 prompt vectors) by incorporating a lightweight and tunable adapter module, in which the intermediate dimension is adjustable, achieving a recognition accuracy of 94.21%. The VPT method, which uses only 64 visual prompt vectors as input, achieves an accuracy of 89.58%, while the FFT method achieves an accuracy of 90.29%.

With the intermediate dimension of the adapter set to 128, Figure 8b presents the recognition accuracy as a function of the number of visual prompt vectors across the four fine-tuning methods. For the VPT method (represented by the red curve), the accuracy reaches its maximum value of 89.58% when the number of prompts is 64. The APT method, based on the AT method with an intermediate dimension of 128, incorporates multiple visual prompt vectors as input. When the number of prompt vectors varies from 1 to 128, the overall accuracy remains relatively stable. With 8 visual prompt vectors, the recognition accuracy reaches 94.75%, surpassing the 93.54% accuracy of the AT method with an intermediate dimension of 128. This demonstrates that the performance of combining both adapters and prompt vectors is superior to using either mechanism alone. With the number of visual prompt vectors set to 8 and the intermediate dimension of the adapter set to 128, the APT method achieved the best recognition rate. Therefore, the same settings will be used for the APT method in the following sections of this paper.

**Figure 9:**
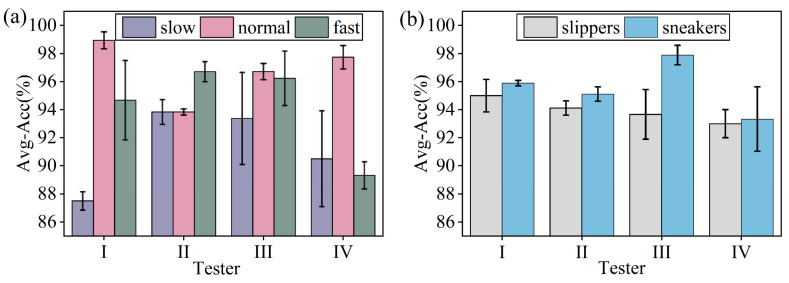
Average test accuracy in the gait recognition task: (**a**) across different walking speeds (slow, normal, fast) for different testers; (**b**) for different testers wearing different footwear (slippers, sneakers). Error bars represent standard deviation over five independent runs.

Figure 9a illustrates the average recognition accuracy of different testers walking at varying speeds (slow, normal, fast). The results show that average recognition accuracy is generally higher at normal walking speed, while slow walking is more challenging to recognize, highlighting the significant impact of walking speed on recognition performance. This can be attributed to the fact that at normal walking speed, gait features are more stable and consistent, leading to clearer and more distinguishable signals. In contrast, slower walking speeds result in less stable gait rhythms, causing the signals to become more ambiguous and reducing recognition accuracy.

Figure 9b presents the average recognition accuracy of four participants wearing different types of shoes. The results indicate that the heaviest participant, Tester III, achieved the highest average recognition accuracy of 97.89% while wearing sneakers, whereas the lightest participant, Tester IV, obtained the lowest average accuracy of 93.33% under the same conditions. Additionally, gait signals recorded while wearing sneakers demonstrated better recognition performance compared to those obtained with slippers. This suggests that sneakers, due to their superior support, stability, and material properties, provide more consistent and clearer gait signals, reducing external interference and improving recognition accuracy. In contrast, slippers, lacking adequate support and stability, result in gait signals that are more susceptible to interference, thereby reducing recognition accuracy. In the testing environment of this study, individuals with higher body weight generate stronger DAS gait signals, leading to higher recognition accuracy when wearing sneakers.

In downstream tasks, fine-tuning is conducted using a small amount of labeled data. The loss function is calculated, and the visual prompt vectors, along with the adapter modules, are updated through stochastic gradient descent (SGD) to improve the model’s performance on the task. Since the parameters of the pre-trained model are kept frozen, the fine-tuning process involves only learning and updating the prompt vectors, the adapter modules, and the classification head. In contrast, FFT requires modifying and storing all parameters of the pre-trained model. For example, the ViT-Base encoder consists of 85,804,808 (85.80 M) parameters, with an image patch embedding dimension of 768 (*d* = 768). Thus, for the MAEPD model based on ViT-Base, the number of trainable parameters under FFT is 85.80 M. For APT, when using eight prompt vectors (*p* = 8) and an adapter with an intermediate dimension of 128 (*q* = 128), the additional trainable parameters are computed as *N* × *p* × *d* + *N* × (2 × *q* × *d* + *q* + *d*) = 2,443,776 (2.44 M), where *N* is the number of transformer encoder layers (*N* = 12). Thus, the updated parameters account for only 2.77% of the total parameters in the frozen encoder, yielding substantial reductions in memory overhead and computational cost. Compared with FFT, the proposed method greatly enhances fine-tuning efficiency. Once the fine-tuning stage is completed, the testing stage is carried out. The preprocessed 2D DAS signals are concatenated with the fine-tuned prompt vectors and fed into the frozen pre-trained model together with the trained adapter modules. Final classification is performed using a classification head.

In the downstream task of gait recognition, we further compared the APT method with baseline approaches including VPT, AT, FFT, and LP in terms of statistical significance. As shown in Table 8, the APT method achieves an average accuracy of 94.75%, which is significantly higher than those of VPT (89.58%), AT (93.54%), FFT (90.29%), and LP (48.21%). Statistical analysis across five independent experimental runs shows that *p*-values for all compared methods are consistently below 0.05. This confirms that the performance advantages of the APT method over other baselines are statistically significant. Although the inference latency of the APT method is 2.74 ms, slightly higher than those of VPT (2.57 ms) and AT (2.60 ms), it outperforms the other methods in terms of average precision (94.76%), average recall (94.75%), and average F1-score (94.75%). Notably, APT updates only 2.44 M parameters, accounting for just 2.77% of the total parameters in the MAEPD model, which represents a 97.16% reduction in trainable parameters compared to the FFT method, along with a 16.67% reduction in training time. In contrast, due to the limited number of training samples (240 per class) in the downstream task, the recognition performance of traditional supervised learning algorithms is notably constrained. As shown in Table 8, the best-performing supervised method, ResNet-18, achieves an average accuracy of only 89.96%, whereas the APT method improves upon this by 4.79 percentage points. Although lightweight networks such as MobileNetV2 achieve the shortest training time of only 1.16 h, their recognition accuracy is merely 88.38%. These results further demonstrate that the APT method achieves a favorable balance among parameter efficiency, training efficiency, and classification performance.

### 5.2. The Impact of Upstream Gait Data Volume on Recognition Performance

Although acquiring large amounts of unlabeled data for upstream pre-training is relatively easy in practical DAS applications, the MAEPD-based foundation model in Section 3 relies on a gait dataset that requires continuous movement and cooperation from participants, making data collection challenging. As a result, only 35,637 samples involving the first six testers in Table 3 were obtained. Therefore, it is essential to investigate the impact of upstream pre-training data volume on model performance, while keeping the training, testing, and validation datasets for downstream tasks in Table 4 unchanged. In this study, we expanded the dataset by incorporating newly collected gait data from the first six participants in Table 3, as well as additional gait data from a specific individual (Tester IV). The expanded gait dataset was then used for upstream pre-training, resulting in an updated model. Subsequently, downstream fine-tuning and testing were conducted using the dataset presented in Table 4.

**Figure 10:**
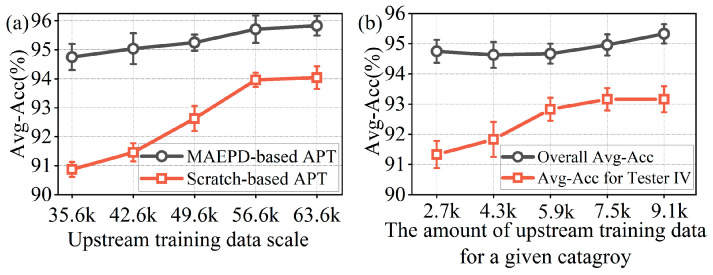
Impact of upstream pre-training data volume on downstream gait average recognition accuracy. (**a**) Comparison of average accuracy versus upstream training data volume between the MAEPD-based APT method and the scratch-based APT method. (**b**) Average accuracy versus the volume of upstream training data for a given class in the MAEPD-based APT method (using only the gait data from Tester IV). Error bars represent standard deviation over five independent runs.

Figure 10a illustrates the trend in average recognition accuracy as the volume of upstream pre-training gait data increases from approximately 35.6 k to 63.6 k samples. To isolate and validate the contribution of the foundation model, we conducted a comparative analysis between the proposed MAEPD-based APT method (fine-tuning the foundation model) and a scratch-based APT baseline (pre-training from scratch using only the corresponding gait data). While both approaches exhibit performance improvements with increased data volume, the MAEPD-based APT consistently outperforms the scratch-based baseline across all data scales. Notably, at the initial scale of 35.6 k samples, the MAEPD-based method achieves an average accuracy of 94.75%, surpassing the 90.87% average accuracy of the scratch-based method. This performance disparity suggests that leveraging diverse pre-training data is valuable. While the scratch-based model relies exclusively on domain-specific gait features, the improved performance of the MAEPD-based model indicates that the inclusion of diverse non-gait data (e.g., seismic, pipeline, and perimeter signals) during pre-training contributes to learning more robust and transferable semantic representations. These findings suggest that the foundation model can effectively utilize multi-domain features to enhance downstream task performance, complementing the use of domain-specific data.

To further investigate the effect of the sample size of a specific category in the upstream training data on downstream recognition performance for that category, Figure 10b illustrates the change in average accuracy when only the additional gait data from Tester IV is added to the original upstream pre-training dataset. The updated model, fine-tuned using APT, was then tested on the downstream task with the dataset presented in Table 4. The results show that as the sample size for this specific category increases, the overall accuracy across all categories improves within the range shown in the figure, while the model’s accuracy for this category in the downstream gait recognition task steadily increases from approximately 91.33% to 93.16%, with a corresponding improvement in overall category recognition. This trend indicates that even a targeted increase in the sample size for a specific category in the upstream data can significantly enhance its performance in downstream tasks. Therefore, increasing the amount and diversity of upstream pre-training data in the future is also a key approach to improving the model’s recognition accuracy.

**Figure 11:**
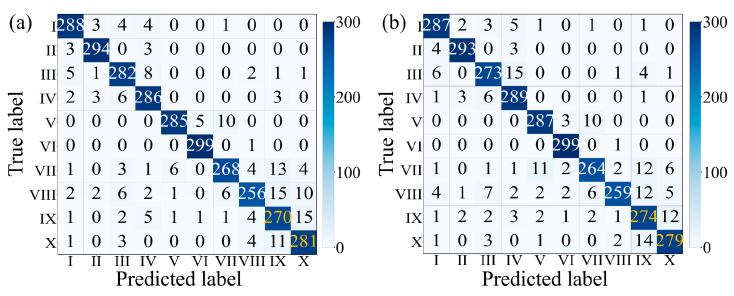
Confusion matrices for gait recognition: (**a**) results obtained using APT on the MAEPD foundation model pre-trained without Tester VII gait data; (**b**) results obtained after updating the foundation model by incorporating an additional 1800 gait samples from Tester VII into the pre-training dataset. The yellow numbers in the confusion matrix indicate the number of correctly classified samples for Tester VII (categories IX and X).

To evaluate the generalization ability of the MAEPD-based foundation model for gait recognition of unfamiliar individuals, an experiment was designed involving a female subject (Tester VII, weight 51 kg). The pre-training data of the MAEPD-based foundation model did not include her gait signals. Compared to other male testers in Table 3, Tester VII has a lighter body weight, resulting in weaker gait signal strength during floor walking. Figure 7g,h show the 2D and 1D gait signals obtained while Tester VII was walking normally and wearing sneakers. 2D spatiotemporal gait signal data were collected from Tester VII in both sneaker and slipper conditions, with 630 samples collected for each condition. Of these, 240 samples were used for downstream fine-tuning, 90 for validation, and 300 for testing. This data was combined with the downstream task dataset in Table 4 for fine-tuning and testing. Tester VII’s gait data was assigned new categories IX (slippers) and X (sneakers). Figure 11a shows the confusion matrix for the model’s performance on Tester VII (categories IX and X), who was not involved in pre-training, as well as on the first four pre-trained participants (Tester I–IV, categories I–VIII). Although Tester VII’s gait data was not included in the upstream pre-training data, the model, after fine-tuning with a small amount of labeled data using the APT method, achieved high classification accuracy, with 90.00% for slippers (category IX) and 93.67% for sneakers (category X). For further comparison, the foundation model was updated by incorporating an additional 1800 gait samples from Tester VII into the pre-training data and then fine-tuned on the same downstream task dataset. As shown in Figure 11b, the corresponding confusion matrix indicates that after updating, the model achieved accuracies of 91.33% for slippers (Category IX) and 93.00% for sneakers (Category X), which are comparable to the results obtained without updating (Figure 11a). These results indicate that even when gait data from subjects not included in the upstream training appears in the downstream task, the MAEPD-based foundation model can still achieve high recognition accuracy through fine-tuning with a limited number of labeled data. This demonstrates, to a certain extent, the effective generalization capability of the proposed foundation model combined with efficient fine-tuning methods for gait recognition involving unseen individuals under the conditions evaluated.

### 5.3. Performance on Other Downstream Tasks

To further evaluate the generalization potential and data efficiency of the proposed MAEPD + APT framework, we conduct experiments on three additional downstream tasks with distinct signal characteristics and application scenarios: water pipe leakage detection, perimeter intrusion detection, and a public perimeter security dataset [53]. The detailed description of each dataset, including preprocessing methods, event categories, and sample splits, has been provided in Section 4.2 and summarized in Table 6. Here, we apply the MAEPD-based APT method (as described in Section 3.2) to each task and compare its performance against several baseline models. All reported results are averaged over five independent runs, with standard deviations included to indicate statistical variability.

Table 9, Table 10 and Table 11 present the performance comparison of various algorithms on the water pipe leakage dataset, the perimeter security detection dataset, and the public DAS perimeter security dataset, respectively, covering recognition performance metrics as well as training time and GPU resource consumption. For the water pipe leakage detection task, when 200 training samples (four classes, 50 samples per class) were used for fine-tuning or training, the data amount for fine-tuning was clearly insufficient, as shown in Table 6. The APT method achieved an average accuracy of 96.26%, outperforming FFT by 1.16%. Moreover, APT updated only 2.44 M parameters and required 0.62 h of training time, still less than the 0.64 h required for FFT. In contrast, supervised algorithms, including MobileNetV2, ResNet-18, ConvNeXt-Tiny, and ViT-Base, performed poorly due to the limited training data. Given the considerable application differences between DAS gait recognition and water pipe leakage detection, this result indicates that the proposed method can maintain strong performance across multiple application scenarios with limited labeled data.

For the perimeter intrusion detection task, as shown in Table 6, a limited number of 125 labeled training samples (five classes, 25 samples per class) were used. As shown in Table 10, APT achieved an average accuracy of 95.58%, significantly surpassing all other methods. For this dataset, the lightweight MobileNetV2 achieved an average recognition rate of 94.45%, with a training time of 0.29 h. Although its accuracy is 1.13% lower than the proposed APT method based on the foundation model, its training time is 0.3 h shorter. While MobileNetV2 performs well on this dataset (Table 10), it performs poorly on the water pipe leakage dataset (Table 9), highlighting that the performance of a single supervised learning algorithm may vary significantly across different datasets.

For the public perimeter security dataset from Beijing Jiaotong University [53], 1200 training samples (six classes, 200 samples per class) were used for fine-tuning, as shown in Table 6. Table 11 shows that APT achieved the highest average accuracy of 96.00%, markedly higher than the best supervised baseline, ResNet-18 (93.13%). For this dataset, ResNet-18 achieved a good recognition rate, further illustrating that the effectiveness of supervised learning algorithms can be dataset-dependent. Overall, results across all datasets consistently demonstrate that the foundation-model-based APT method delivers superior recognition performance compared to other methods, while maintaining a favorable balance in training time and GPU resource consumption. Compared to supervised learning methods, the foundation-model-based APT method achieves the highest recognition rates across several datasets with limited labeled data, whereas supervised algorithms tend to show inconsistent performance across different datasets.

**Figure 12:**
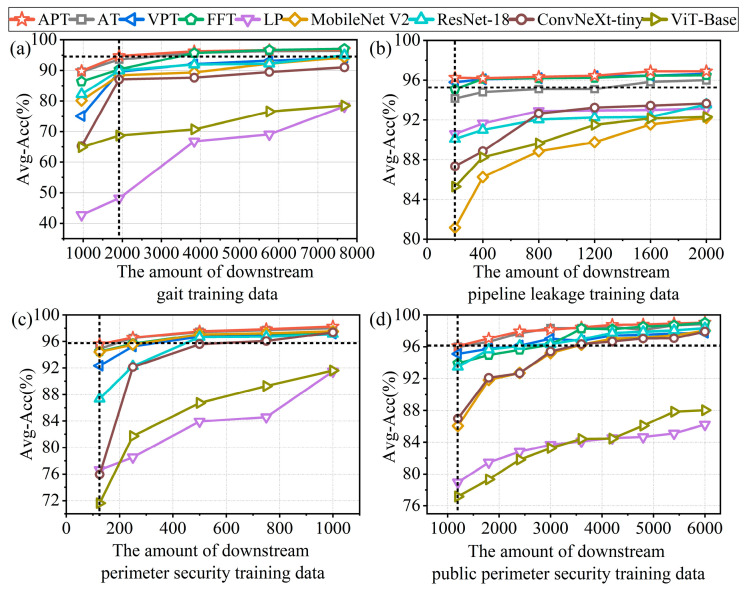
Performance as a function of fine-tuning dataset size using the proposed method and baseline algorithms on (**a**) the gait dataset, (**b**) the water pipe leakage dataset, (**c**) the perimeter security dataset, and (**d**) the third-party public perimeter security dataset [53]. The dashed vertical lines indicate the specific training dataset sizes used for the compared methods in Table 8, Table 9, Table 10 and Table 11, respectively.

Subsequently, we further investigate the dependence of various downstream tasks on the amount of downstream training data, including the gait signal dataset in Section 4, the water pipe leakage dataset, the perimeter security dataset, and the third-party public perimeter security dataset [53]. Using the proposed method and multiple baseline algorithms, we systematically analyze the recognition accuracy of each downstream task as the fine-tuning training data size changes. This provides a comprehensive assessment of the performance of different methods under limited labeled data conditions, as well as their sensitivity to data size and adaptability to different datasets. Figure 12a–d illustrate the recognition performance as the fine-tuning training dataset size changes, using the proposed MAEPD-based APT method and multiple baseline algorithms on the gait dataset, water pipe leakage dataset, perimeter security dataset, and third-party public perimeter security dataset [53]. As shown in Figure 12a, in the gait recognition task, when the fine-tuning training dataset size increased from 1920 to 5760, the accuracy of MobileNetV2 and ResNet-18 improved from 88.38% to 94.17% and from 89.96% to 95.00%, respectively. In contrast, the accuracy of the foundation-model-based APT method only slightly increased, from 94.75% to 96.67%. These results indicate that the APT method is less sensitive to training data size, maintaining high accuracy even with limited labeled data. Overall, the MAEPD-based fine-tuning method, except for the LP method (which performs poorly due to training only the linear classifier), shows good performance across other fine-tuning algorithms, with relatively low sensitivity to data size. Several supervised algorithms, however, appear more sensitive to data size, with their performance approaching that of fine-tuning methods when ample training data is available. Similar trends are observed in the other three datasets shown in Figure 12b–d. The fine-tuning methods based on foundation models and the supervised learning algorithms exhibit varying degrees of sensitivity to the fine-tuning data size. These results suggest that the foundation-model-based methods, having learned to extract relevant task-specific semantic information during upstream pre-training, can maintain high recognition accuracy with fewer labeled data in downstream tasks. This characteristic is especially suitable for practical DAS systems, where large amounts of unlabeled data are often available, but labeled data are scarce. While some supervised algorithms perform well on individual datasets with limited labeled data, their effectiveness does not consistently generalize across different datasets. For example, ResNet-18 performs reasonably well on the public perimeter security dataset but shows lower accuracy on the in-house perimeter security dataset. Under limited labeled data conditions, the proposed MAEPD-based APT method achieves the highest recognition accuracy among the compared methods across all four datasets evaluated, demonstrating consistent multi-scenario performance and efficient use of labeled data. Taking the recognition results from the publicly available third-party dataset [53] shown in Figure 12d as an example, the APT method achieves a recognition accuracy of 96.00% using only 1200 training samples. In comparison, methods such as AT, VPT, FFT, ResNet-18, ConvNeXt-Tiny, and MobileNetV2 require approximately 1300, 2200, 2800, 2300, 3400, and 3500 training samples, respectively, to achieve or approach the same accuracy. Compared to the well-performing supervised algorithm ResNet-18, the APT method reduces the training data requirement by 47.83%.

As shown in Table 12, the proposed APT method significantly outperforms the best traditional baselines across all four downstream tasks, achieving accuracy improvements of 4.79%, 5.16%, 1.13%, and 2.87%, respectively. In addition, Table 13 presents a comparative analysis between the MAEPD + APT framework and representative DAS recognition methods reported in the literature. For the gait recognition task, traditional CNN and YOLO models, which require full parameter updates (100%), achieve accuracies of 81.35% and 86.0%, respectively. In contrast, our method attains a higher accuracy of 94.75% while updating only 2.77% of the parameters. Even when compared with the ConvNeXt model, which performs well in perimeter security with 99.3% accuracy but requires 1080 training samples per class, our method maintains a competitive accuracy of 95.58% under the extremely limited data scenario of using only 25 samples per class. These results demonstrate its generalization capability under limited training sample conditions. In summary, despite notable differences in experimental equipment, data collection environments, and event types across the four datasets, the MAEPD-based APT method demonstrates competitive recognition performance in several aspects: (1) Multi-scenario applicability: Pre-training on large-scale DAS data enables the model to extract relevant signal features and adapt to various task requirements, reducing the need for training from scratch; (2) Label efficiency: The model achieves strong performance with relatively limited labeled data during fine-tuning; (3) Reduced data dependency: By leveraging pre-trained knowledge, the model can effectively utilize upstream representations to support downstream tasks with fewer labeled examples. These results suggest that incorporating diverse data during upstream pre-training can help achieve reasonable recognition performance in downstream tasks with limited labeled data. However, several limitations of the current study should be acknowledged. First, incorporating broader unlabeled data from more diverse real-world DAS measurement scenarios and from different DAS devices may further enhance the robustness and generalization capability of the model. Second, although the APT method reduces trainable and storable parameters, there remains room for optimizing inference latency in practical deployment scenarios.

## 6. Conclusions

This paper proposes a DAS signal recognition method based on self-supervised learning, aimed at enabling effective recognition across multiple DAS measurement scenarios. First, a large-scale, diverse image dataset covering several typical DAS application domains is used to pre-train a self-supervised masked autoencoder (MAE), resulting in a DAS-specific foundation model, termed MAEPD. Through this pre-training, the MAEPD encoder captures general semantic information from DAS signals. Next, the adapter-based prompt tuning (APT) method is applied for downstream fine-tuning, reducing computational and storage overhead as well as training time. Experimental results show that, for gait signal recognition, the APT method achieves an accuracy of 94.75% with only 240 images per class, outperforming full fine-tuning by 4.46%. Moreover, APT updates only 2.77% of the parameters, reduces training time by 16.67%, and lowers GPU memory consumption. Compared to supervised learning algorithms, this method reduces the amount of labeled data required for training, while demonstrating competitive performance across the four datasets evaluated in this study, including gait recognition, pipeline leakage detection, and perimeter security events. These results further suggest its potential for various downstream tasks. Overall, the MAEPD foundation model combined with APT fine-tuning provides an efficient and scalable approach to address challenges such as model generalization and training costs in DAS measurements, contributing to the development of DAS signal recognition methods for diverse applications.

## Figures and Tables

**Table 1:** Composition of the MAEPD pre-training dataset.

Dataset	Source	Input Size	Preprocessing Method	Number of Samples	Purpose
Gait	Self-collected	(100,000 sample points, 8 channel)	Spatiotemporal stacking	35,637	Pre-training
Pipeline Leakage	Self-collected	(4000 sample points, 1 channel)	STFT	260,154	Pre-training
Perimeter Intrusion 1	Self-collected	(20,000 sample points, 1 channel)	GASF	150,170	Pre-training
Perimeter Intrusion 2	Public [53]	(10,000 sample points, 12 channel)	Spatiotemporal stacking	6000	Pre-training
Whale Vocalization	Public [54]	(12,000 sample points, 40 channel)	Spatiotemporal stacking	171,302	Pre-training
Seismic	Public [55]	(75,000 sample points, 40 channel)	Spatiotemporal stacking	12,597	Pre-training
Total	/	/	/	635,860	/

**Table 2:** Hyperparameters and experimental setup for MAEPD pre-training.

Configuration Item	Setting
Input image, Patch size and Number of patches	224 × 224, patch = 16 × 16, *N* = 196
Masking ratio	75% random masking
Encoder architecture	ViT-Base (12 transformer layers, *d* = 768)
Decoder architecture	8 transformer layers
Initial learning rate and Optimizer	0.001, AdamW
Learning rate schedule	Cosine annealing
Total training time	350 h and 20 min
Final training loss	0.020 (converged around epoch 200)

**Table 3:** Several gait-related physiological parameters of seven volunteers.

Tester	Sex	Weight (kg)
Tester I	Male	75
Tester II	Male	72
Tester III	Male	80
Tester IV	Male	60
Tester V	Male	80
Tester VI	Male	65
Tester VII	Female	51

**Table 4:** Multiple DAS gait categories and their corresponding data quantities for downstream tasks.

Type	Event	Class	Walking Statuses	Train/Val/Test	Num
Downstream Task Dataset	Tester I-Slippers	I	Slow Walking	80/30/100	210
			Normal Walking	80/30/100	210
			Fast Walking	80/30/100	210
	Tester I-Sneakers	II	Slow Walking	80/30/100	210
			Normal Walking	80/30/100	210
			Fast Walking	80/30/100	210
	Tester II-Slippers	III	Slow Walking	80/30/100	210
			Normal Walking	80/30/100	210
			Fast Walking	80/30/100	210
	Tester II-Sneakers	IV	Slow Walking	80/30/100	210
			Normal Walking	80/30/100	210
			Fast Walking	80/30/100	210
	Tester III-Slippers	V	Slow Walking	80/30/100	210
			Normal Walking	80/30/100	210
			Fast Walking	80/30/100	210
	Tester III-Sneakers	VI	Slow Walking	80/30/100	210
			Normal Walking	80/30/100	210
			Fast Walking	80/30/100	210
	Tester IV-Slippers	VII	Slow Walking	80/30/100	210
			Normal Walking	80/30/100	210
			Fast Walking	80/30/100	210
	Tester IV-Sneakers	VIII	Slow Walking	80/30/100	210
			Normal Walking	80/30/100	210
			Fast Walking	80/30/100	210

**Table 5:** Comparison of estimated walking speeds and actual values for different walking patterns.

Tester	Walking Statuses	Calculated Speed (m/s)	Ground Truth (m/s)
Tester I	Slow	0.52	0.44
	Normal	0.71	0.8
	Fast	0.83	0.87
Tester II	Slow	0.42	0.51
	Normal	0.54	0.52
	Fast	0.88	0.86
Tester III	Slow	0.55	0.56
	Normal	0.74	0.82
	Fast	0.95	0.9
Tester IV	Slow	0.48	0.42
	Normal	0.58	0.55
	Fast	0.71	0.64

**Table 6:** Categories and sample quantities for the downstream tasks in the pipeline leakage dataset, the perimeter intrusion dataset, and the publicly available third-party perimeter security dataset.

Type	Preprocessing Method	Event	Train/Val/Test	Num
Water Pipe Leakage Dataset	STFT	Leakage	50/100/500	650
		No Leakage	50/100/500	650
		Hammering	50/100/500	650
		Cutting	50/100/500	650
Perimeter Intrusion Dataset	Spatiotemporal stacking	Shaking	25/100/300	425
		UAV intrusion	25/100/300	425
		Biting	25/100/300	425
		Trampling	25/100/300	425
		Cutting	25/100/300	425
Public Perimeter Security Dataset [53]	Spatiotemporal stacking	Shaking	200/100/546	846
		Digging	200/100/502	802
		Knocking	200/100/506	806
		Walking	200/100/490	790
		Watering	200/100/451	751
		Background	200/100/588	888

**Table 7:** Hyperparameter optimization of different methods for gait recognition.

Title	MAEPD Based Fine-Tuning			Traditional Supervised Learning Models
Method	FFT or LP	VPT	AT, APT	MobileNetV2, ResNet-18, ConvNext-Tiny, ViT-Base
Optimizer	SGD	SGD	SGD	SGD
Momentum	0.9	0.9	0.9	0.9
base_lr range	[0.0001,0.005]	[0.05,5]	[0.05,5]	[0.0001,0.005]
Weight decay	[0,0.01]	[0,0.01]	[0,0.01]	[0,0.01]
Scale	/	/	[0.01,0.1]	/
lr schedule	Cosine	Cosine	Cosine	Cosine
Epochs	300	300	300	300

**Table 8:** A comparison of the performance, tunable parameters, and training time consumption between different fine-tuning algorithms and supervised learning algorithms.

Method	Accuracy (%)	Avg-Precision (%)	Avg-Recall (%)	Avg-F1 Score (%)	*p*-Value (vs. APT)	Tunable Parameter (M)	Train Time (h)	Inference Time (ms)
APT	94.75 ± 0.22	94.76 ± 0.22	94.75 ± 0.22	94.75 ± 0.22	/	2.44	1.75	2.74
AT	93.54 ± 0.29	93.58 ± 0.30	93.54 ± 0.29	93.54 ± 0.29	0.0090	2.27	1.67	2.60
VPT	89.58 ± 0.32	89.61 ± 0.36	89.58 ± 0.32	89.57 ± 0.33	<0.05	0.59	1.41	2.57
FFT	90.29 ± 0.29	90.39 ± 0.36	90.29 ± 0.29	90.30 ± 0.31	<0.05	85.80	2.10	2.34
LP	48.21 ± 0.40	47.89 ± 0.49	48.21 ± 0.40	46.81 ± 0.60	<0.05	0.0061	1.25	2.34
MobileNetV2	88.38 ± 0.63	88.39 ± 0.71	88.38 ± 0.63	88.31 ± 0.66	<0.05	2.23	1.16	1.46
ResNet-18	89.96 ± 1.73	90.26 ± 1.31	89.96 ± 1.73	89.93 ± 1.40	<0.05	11.18	1.20	1.48
ConvNeXt	87.08 ± 2.10	87.22 ± 2.05	87.08 ± 2.10	87.02 ± 2.12	<0.05	27.82	1.24	2.08
ViT-Base	68.70 ± 0.82	68.43 ± 0.79	68.70 ± 0.82	68.13 ± 0.92	<0.05	85.80	2.10	2.34

**Table 9:** Performance comparison of different algorithms on the water pipe leakage dataset, including evaluation metrics and time/GPU resource consumption.

Method	Accuracy (%)	Avg-Precision (%)	Avg-Recall (%)	Avg-F1 Score (%)	*p*-Value (vs. APT)	Tunable Parameter (M)	Train Time (h)	Inference Time (ms)
APT	96.26 ± 0.24	96.60 ± 0.24	96.15 ± 0.24	96.15 ± 0.24	/	2.44	0.62	2.83
AT	94.16 ± 0.19	94.16 ± 0.26	94.16 ± 0.19	94.08 ± 0.20	<0.05	2.27	0.55	2.70
VPT	95.80 ± 0.11	96.02 ± 0.12	95.80 ± 0.11	95.79 ± 0.11	0.0093	0.59	0.47	2.69
FFT	95.10 ± 0.50	95.50 ± 0.36	95.10 ± 0.11	95.08 ± 0.46	<0.05	85.80	0.64	2.42
LP	90.59 ± 0.40	91.39 ± 0.34	90.59 ± 0.40	90.33 ± 0.37	<0.05	0.0030	0.40	2.42
MobileNetV2	81.15 ± 0.66	81.32 ± 0.53	81.15 ± 0.66	81.07 ± 0.58	<0.05	2.22	0.37	1.48
ResNet-18	91.10 ± 1.34	91.25 ± 1.20	91.10 ± 1.34	91.11 ± 1.32	<0.05	11.17	0.38	1.45
ConvNeXt	87.34 ± 0.56	87.42 ± 0.48	87.34 ± 0.56	87.25 ± 0.53	<0.05	27.82	0.47	2.14
ViT-Base	85.29 ± 0.43	85.30 ± 0.66	85.29 ± 0.43	84.90 ± 0.79	<0.05	85.80	0.64	2.42

**Table 10:** Performance comparison of different algorithms on the perimeter security detection dataset, including evaluation metrics and time/GPU resource consumption.

Method	Accuracy (%)	Avg-Precision (%)	Avg-Recall (%)	Avg-F1 Score (%)	*p*-Value (vs. APT)	Tunable Parameter (M)	Train Time (h)	Inference Time (ms)
APT	95.58 ± 0.26	95.62 ± 0.24	95.58 ± 0.26	95.58 ± 0.26	/	2.44	0.59	2.60
AT	94.90 ± 0.23	95.01 ± 0.20	94.90 ± 0.23	94.90 ± 0.24	0.0024	2.27	0.53	2.49
VPT	92.34 ± 0.46	92.62 ± 0.40	92.34 ± 0.46	92.35 ± 0.48	<0.05	0.59	0.47	2.47
FFT	94.50 ± 0.32	94.62 ± 0.33	94.50 ± 0.32	94.52 ± 0.31	<0.05	85.80	0.61	2.16
LP	76.61 ± 1.10	77.83 ± 0.88	76.61 ± 1.10	76.27 ± 1.10	<0.05	0.0038	0.37	2.16
MobileNetV2	94.45 ± 0.39	94.61 ± 0.36	94.45 ± 0.39	94.41 ± 0.29	<0.05	2.23	0.29	1.28
ResNet-18	87.36 ± 0.27	87.60 ± 0.39	87.36 ± 0.27	87.23 ± 0.28	<0.05	11.17	0.32	1.34
ConvNeXt	75.93 ± 0.24	76.70 ± 0.76	75.93 ± 0.24	75.72 ± 0.24	<0.05	27.82	0.46	1.99
ViT-Base	71.59 ± 0.37	72.63 ± 0.42	71.59 ± 0.37	71.43 ± 0.36	<0.05	85.80	0.61	2.16

**Table 11:** Performance comparison of different algorithms on the public DAS perimeter security dataset [53], including evaluation metrics and time/GPU resource consumption.

Method	Accuracy (%)	Avg-Precision (%)	Avg-Recall (%)	Avg-F1 Score (%)	*p*-Value (vs. APT)	Tunable Parameter (M)	Train Time (h)	Inference Time (ms)
APT	96.00 ± 0.37	95.98 ± 0.48	96.00 ± 0.37	95.98 ± 0.52	/	2.44	1.04	2.71
AT	95.39 ± 0.45	95.40 ± 0.45	95.39 ± 0.45	95.38 ± 0.45	0.038	2.27	1.03	2.64
VPT	94.82 ± 0.40	94.87 ± 0.38	94.82 ± 0.40	94.82 ± 0.40	<0.05	0.59	1.01	2.64
FFT	93.90 ± 0.42	93.90 ± 0.14	93.90 ± 0.42	93.88 ± 0.35	<0.05	85.80	1.11	2.33
LP	77.20 ± 2.47	77.16 ± 2.77	77.20 ± 2.47	77.68 ± 2.34	<0.05	0.0046	0.92	2.33
MobileNetV2	86.43 ± 0.28	86.92 ± 0.12	86.43 ± 0.28	86.40 ± 0.29	<0.05	2.23	0.51	1.43
ResNet-18	93.13 ± 0.27	93.25 ± 0.27	93.13 ± 0.27	93.15 ± 0.26	<0.05	11.18	0.53	1.48
ConvNeXt	86.95 ± 0.23	86.95 ± 0.39	86.95 ± 0.23	87.00 ± 0.29	<0.05	27.8	0.67	2.03
ViT-Base	77.21 ± 0.45	77.16 ± 0.66	77.21 ± 0.42	77.13 ± 0.56	<0.05	85.80	1.11	2.33

**Table 12:** Summary of Performance Comparisons on Four DAS Downstream Tasks.

Dataset	Training Samples (per Class)	Best Traditional Supervised Baseline (Avg-Acc %)	Proposed APT (Avg-Acc %)	Accuracy Gain (%)
Gait Recognition	240	89.96 (ResNet-18)	94.75	+4.79
Water Pipe Leakage	50	91.10 (ResNet-18)	96.26	+5.16
Perimeter Intrusion	25	94.45 (MobileNetV2)	95.58	+1.13
Public Dataset [53]	200	93.13 (ResNet-18)	96.00	+2.87

**Table 13:** Performance comparison of the proposed MAEPD + APT method with representative DAS signal recognition approaches reported in the literature.

Core Idea	Application Scenario	Training Data (per class)	Test Accuracy (%)	Parameter Update (%)
CNN-LSTM [26]	perimeter security	Not specified (3 class)	97.3	100
Transformer [29]	power cable intrusion	2000 (3 class)	97.3	100
AlexNet [31]	perimeter security	372(8 class)	96.16	100
ConvNext [37]	perimeter security	1080(6 class)	99.3	100
CNN [60]	gait recognition	71 (2 class)	81.35	100
YOLO [61]	gait recognition	450(3 class)	86	100
MAEPD + APT (Ours)	gait/leakage/perimeter	240/50/25 (8,4,5 class)	94.75/96.26/95.58	2.77

**Figure A1:**
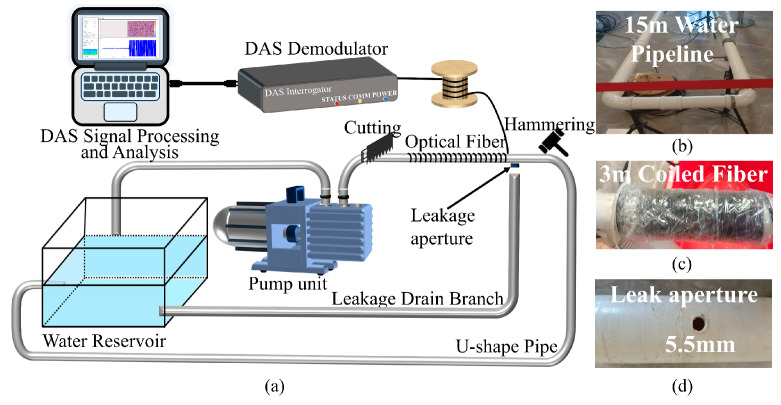
(**a**) The schematic diagram of the experimental setup for the DAS water pipe leakage monitoring system. (**b**) Photograph of the PVC U-shaped pipe. (**c**) Photograph of the armored fiber optic cable (black) wrapped around the outer surface of the pipe at the leakage point. (**d**) Image of the leakage hole in the PVC pipe, with a hole diameter of approximately 5.5 mm.

**Figure A2:**
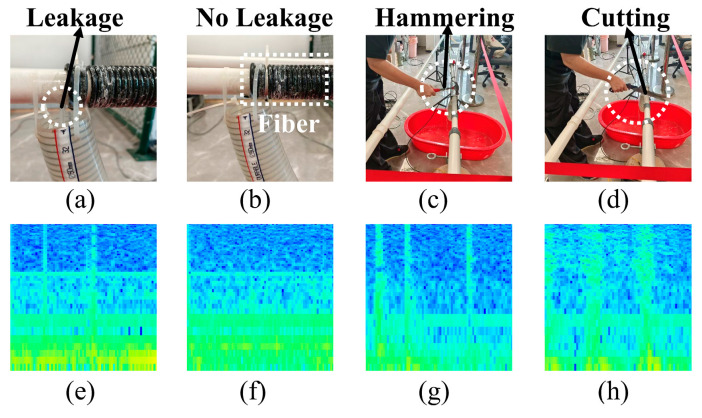
Images of the actual scenarios for the four simulated water pipe leakage events: (**a**) Leakage, (**b**) No leakage, (**c**) Hammering, (**d**) Cutting. Time–frequency image signals obtained for the four events, where the horizontal axis represents time and the vertical axis represents frequency: (**e**) Leakage, (**f**) No leakage, (**g**) Hammering, (**h**) Cutting.

**Figure A3:**
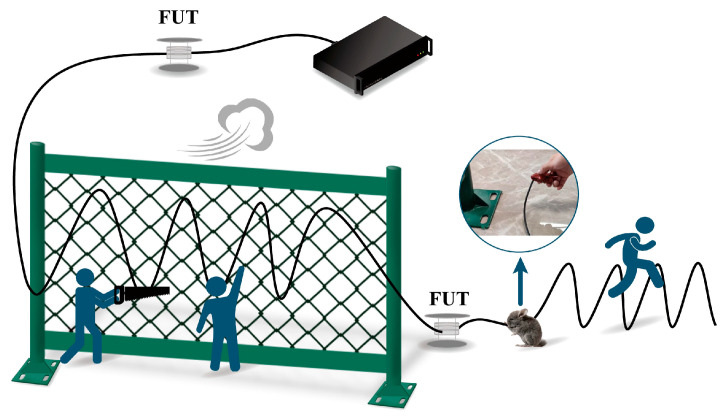
The schematic diagram of the experimental setup for the DAS-based perimeter security system.

**Figure A4:**
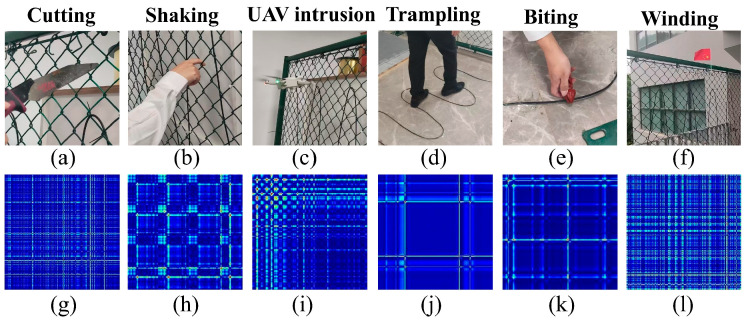
Images of the actual scenarios for the six simulated perimeter security events: (**a**) Cutting, (**b**) Shaking, (**c**) UAV intrusion, (**d**) Trampling, (**e**) Biting, (**f**) Winding. 2D GASF image signals obtained for the six events: (**g**) Cutting, (**h**) Shaking, (**i**) UAV intrusion, (**j**) Trampling, (**k**) Biting, (**l**) Winding.

**Figure A5:**
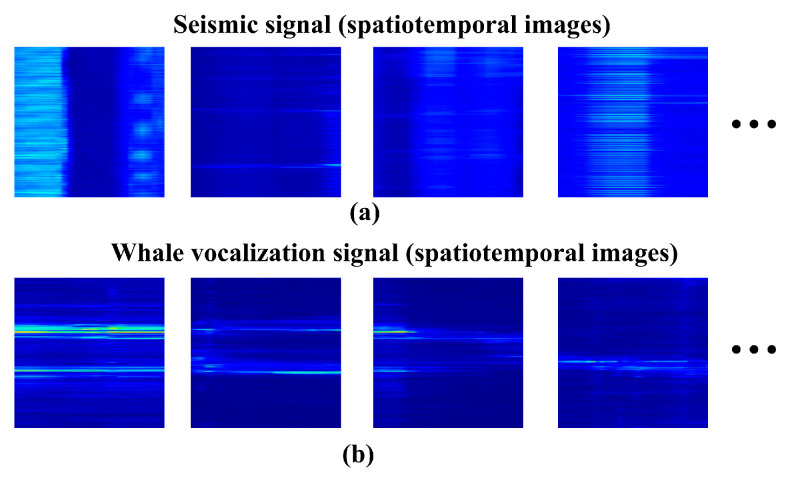
DAS 2D spatiotemporal image data obtained from partially publicly available datasets: (**a**) Seismic signal [55]. (**b**) Whale vocalization signal [54].

**Figure A6:**
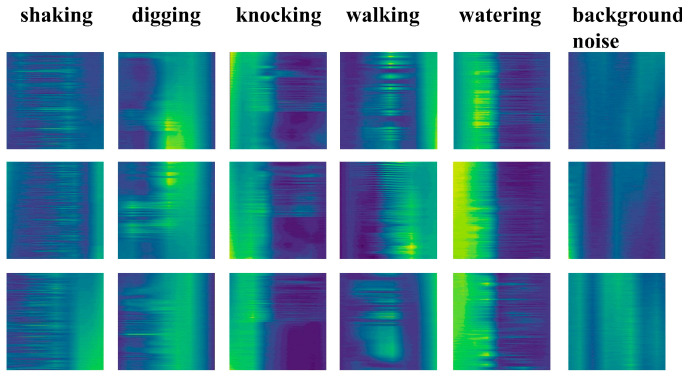
DAS 2D spatiotemporal image data for perimeter security, obtained from partially publicly available datasets [53]. The dataset includes six event types: shaking, digging, knocking, walking, watering, and background noise.

## Data Availability

Data is available upon reasonable request to the corresponding author.

## Abbreviations

The following abbreviations are used in this manuscript:

DAS	Distributed Acoustic Sensing
AI	Artificial Intelligence
φ-OTDR	Phase-sensitive Optical Time-Domain Reflectometry
NLL	Narrow Linewidth Laser
AOM	Acousto-Optic Modulator
EDFA	Erbium-doped Optical Fiber Amplifier
BPD	Balanced Photodetector
ADC	Analog-to-Digital Converter
LO	Local-Oscillator
I/Q	In-phase/Quadrature
MAE	Masked Autoencoder
MAEPD	Masked Autoencoder Pre-training on DAS signals
APT	Adapter-based Prompt Tuning
VPT	Visual Prompt Tuning
AT	Adapter Tuning
FFT	Full Fine-Tuning
LP	Linear Probing
ViT	Vision Transformer
CNN	Convolutional Neural Network
LSTM	Long Short-Term Memory
CBAM	Convolutional Block Attention Module
MHSA	Multi-head Self-Attention
MLP	Multi-Layer Perceptron
SGD	Stochastic Gradient Descent
MSE	Mean Squared Error
STFT	Short-Time Fourier Transform
GAF	Gramian Angular Fields
GASF	Gramian Angular Summation Fields
1D	One-dimensional
2D	Two-dimensional
3D	Three-dimensional
GS	Gait Signals
PLS	Pipeline-Leakage Signals
PIS	Perimeter-Intrusion Signals
WVS	Whale Vocalization Signals
SS	Seismic Signals
PDA	Partial Domain Adaptation

## Appendix A

### Appendix A.1. A Detailed Introduction to the Experimental Scenario and Dataset for Water Pipe Leakage Detection

The water pipe leakage monitoring experiment is conducted in a controlled environment that simulates real-world conditions. The schematic diagram of the experimental setup for the DAS water pipe leakage monitoring system, as shown in Figure A1, consists of a water reservoir, pump, U-shaped main pipeline, sensing fiber optic cable, DAS demodulator, and industrial control computer. A 750 W pump extracts water from the reservoir to provide circulating power for the system. The U-shaped main pipeline serves as the backbone for water circulation, with water extracted by the pump flowing through the pipe and returning to the reservoir via a hose, forming a closed-loop circulation. The pump is located outdoors to minimize interference from its operational noise. The test pipe is a 32 mm radius PVC pipe, with leakage holes drilled at specific diameters, and fitted with removable rubber plugs for quick switching between “leakage” and “non-leakage” conditions. A leakage branch is installed beneath the leakage hole to guide the leaked water back to the reservoir, ensuring both safety and circulation efficiency. The armored fiber optic cable is tightly wound in a spiral pattern around the outer wall of the test section and is double-secured with epoxy resin and adhesive tape, ensuring effective vibration detection on the inner pipe wall. The DAS demodulator has a spatial resolution of 10 m and a sampling rate of 2 kS/s, continuously collecting one-dimensional (1D) raw amplitude signals, which are transmitted to the industrial control computer for processing via a gigabit Ethernet connection.

In the laboratory, several “events” were simulated for water pipe leakage detection. The first event involved sealing the leakage hole to test the scenario with no leakage, referred to as the “no leakage” event. The second event involved removing the plug to allow leakage, and to prevent the sound of falling water from affecting the measurements, the leaked water was recovered and silenced using a hose. The third and fourth events involved lightly striking or cutting the pipe approximately 30 cm from the leakage point, referred to as “hammering” and “cutting” events, respectively. The actual scenes of these four events are shown in Figure A2a–d. The collected 2 s 1D amplitude signals were converted into two-dimensional (2D) time–frequency images using short-time Fourier transform (STFT) [49,50], as shown in Figure A2e–h. To introduce signal diversity, the distance between the DAS demodulator and the leakage point was varied (25 km, 35 km and 45 km), along with changes in water pressure (from 0.05 MPa, 0.1 MPa and 0.15 MPa), leakage hole size (2 mm, 4 mm and 5.5 mm), and indoor noise levels (18 dB, 42 dB, and 75 dB). Measurements were taken at different times, resulting in a total of 262,754 224 × 224 time–frequency image samples. Among these, 260,154 samples were used for upstream pre-training (without labels), and 2600 samples were used for downstream task, with 200 samples allocated for training, 400 for validation, and 2000 for testing.

### Appendix A.2. A Detailed Introduction to the Experimental Scenario and Dataset for Perimeter Security Detection

In the laboratory, a perimeter security scenario was established using a DAS demodulator and armored fiber optic cable to collect amplitude signals from natural wind and various human intrusion events. The schematic diagram of the perimeter security experimental setup is shown in Figure A3. Two typical perimeter security scenarios were created: first, the S-shaped armored fiber optic cable was suspended on a fence to simulate intrusion or damage to the fence; second, the fiber optic cable was laid directly on the ground to simulate footstep intrusion. The DAS demodulator has a spatial resolution of 5 m and a sampling rate of 20 kS/s, and is connected to an industrial control computer via a gigabit Ethernet card to collect 1D amplitude signals from the corresponding channels.

In the experiment, multiple perimeter intrusion events were simulated: the cutting event simulated an intruder using a saw to directly damage the fence; the shaking event involved applying external force to shake the fence; the unmanned aerial vehicle (UAV) event simulated a small UAV (DJI Mini 4 Pro, DJI, Shenzhen, China) flying low and approaching the fence; the trampling event involved an experimenter stepping on the fiber optic cable laid on the ground; the animal biting event used a simulated animal toy’s teeth to bite the fiber optic cable, simulating the scenario of wildlife biting the cable outdoors; the natural wind event involved placing a section of the fence outdoors to directly capture signals from the wind. To effectively highlight key features within complex interference signals, the Gramian angular summation field (GASF) method was applied to convert the 1D amplitude signal into a feature-rich 2D image [37]. As shown in Figure A4a–f represent the actual scenarios of the cutting, shaking, UAV intrusion, trampling, animal biting, and natural wind events, while Figure A4g–l show their corresponding 2D GASF images. To introduce signal diversity, the experiment varied the distance between the event location and the demodulator (0.35 km, 2 km and 4 km), used different experimenters to collect signals, and changed the indoor noise level (16 dB, 40 dB and 70 dB). Measurements were taken at different times, resulting in a total of 152,295 224 × 224 2D GASF image samples. Of these, 150,170 samples were used for upstream pre-training (without labels), and 2125 samples were used for downstream task, with 125 samples allocated for training, 500 for validation, and 1500 for testing.

### Appendix A.3. Introduction to Other Datasets Used for the Foundation Model

The seismic data in this study were collected through the Penn State Fiber Optic for Environmental Sensing (FORESEE) project [55]. The experiment utilized DAS technology, monitoring nearly 5 km of pre-existing dark telecommunication fiber beneath the Penn State campus. The primary objective of the experiment was to study urban geohazards and hydrological systems through near-surface seismic monitoring. Key findings from the data analysis include the successful capture of broadband seismic signals, demonstrating that DAS can effectively detect various seismic sources. These signals include natural seismic events (such as global and regional earthquakes) as well as anthropogenic sources (such as mining blasts and even live music performances). Additionally, the study provides a detailed calibration procedure, converting DAS data into meaningful seismic measurements and comparing them with traditional seismometer data. All DAS recorded data are stored in TDMS format, the default data format for the Silixa iDAS system. Finally, a portion of the DAS data was cropped into 12,597 spatiotemporal image samples of 224 × 224, as shown in Figure A5a, where the horizontal axis represents time and the vertical axis represents the channel number.

The DAS data for whale vocalizations were collected during the maintenance shutdown of the Ocean Observatories Initiative (OOI) cabled observatory systems from 1 to 5 November 2021, using two OptaSense QuantX systems [54]. Data acquisition took place on the receive and transmit fibers at the OOI shore station in Pacific City, Oregon. The data were recorded in three primary configurations, each with varying fiber lengths, sampling frequencies, and gauge lengths to meet different experimental requirements. For example, one configuration used a 95 km fiber, 2 m channel spacing, 47,500 channels, and a sampling frequency of 200 Hz. The DAS data were stored in HDF5 (H5) format, with recording intervals of 30 or 60 s. These data files contain optical phase shift information, which is directly proportional to strain, allowing for the capture of low-frequency signals. The collected data includes various types of signals, such as natural events (e.g., earthquakes, whale vocalizations) and anthropogenic activities (e.g., vessel movements, mining blasts). The data captures a broad frequency range, including fin whale vocalizations, blue whale signals, and an earthquake event from Northern California. This dataset provides comprehensive seismic and acoustic data suitable for monitoring and analyzing geophysical and marine phenomena using DAS technology. Ultimately, a portion of the DAS data was cropped into 171,302 spatiotemporal image samples of 224 × 224, as shown in Figure A5b, where the horizontal axis represents time and the vertical axis represents channel number.

The third dataset is a publicly available DAS perimeter security dataset collected by Yu’s group from the School of Computer and Information Technology at Beijing Jiaotong University [53]. This dataset includes six types of events, background noise, digging, knocking, watering, shaking, and walking, with each event type assigned a unique label (0–5). The dataset contains a total of 15,612 samples, with corresponding data for each event type. Each sample is cropped into a spatiotemporal signal consisting of 10,000 time-domain points and 12 adjacent spatial points. The data were collected under different conditions, with varying recording durations and locations for each event type. From the full dataset, 10,883 samples were further processed into 224 × 224 spatiotemporal images, as shown in Figure A6, where the horizontal axis represents the channel number and the vertical axis represents time. The dataset is divided into three parts: 6000 images for upstream pre-training, and 4883 samples were used for downstream task, with 1200 samples allocated for training, 600 for validation, and 3083 for testing.
